# Multivariate matching pursuit in optimal Gabor dictionaries: theory and software with interface for EEG/MEG via Svarog

**DOI:** 10.1186/1475-925X-12-94

**Published:** 2013-09-23

**Authors:** Rafał Kuś, Piotr Tadeusz Różański, Piotr Jerzy Durka

**Affiliations:** 1 Faculty of Physics, University of Warsaw, ul. Hoża 69, 00-681 Warszawa, Poland

**Keywords:** Matching pursuit, EEG, MEG, Time-frequency, Gabor dictionary, Metrics

## Abstract

**Background:**

Matching pursuit algorithm (MP), especially with recent multivariate extensions, offers unique advantages in analysis of EEG and MEG.

**Methods:**

We propose a novel construction of an optimal Gabor dictionary, based upon the metrics introduced in this paper. We implement this construction in a freely available software for MP decomposition of multivariate time series, with a user friendly interface via the Svarog package (Signal Viewer, Analyzer and Recorder On GPL, http://braintech.pl/svarog), and provide a hands-on introduction to its application to EEG. Finally, we describe numerical and mathematical optimizations used in this implementation.

**Results:**

Optimal Gabor dictionaries, based on the metric introduced in this paper, for the first time allowed for *a priori* assessment of maximum one-step error of the MP algorithm. Variants of multivariate MP, implemented in the accompanying software, are organized according to the mathematical properties of the algorithms, relevant in the light of EEG/MEG analysis. Some of these variants have been successfully applied to both multichannel and multitrial EEG and MEG in previous studies, improving preprocessing for EEG/MEG inverse solutions and parameterization of evoked potentials in single trials; we mention also ongoing work and possible novel applications.

**Conclusions:**

Mathematical results presented in this paper improve our understanding of the basics of the MP algorithm. Simple introduction of its properties and advantages, together with the accompanying stable and user-friendly Open Source software package, pave the way for a widespread and reproducible analysis of multivariate EEG and MEG time series and novel applications, while retaining a high degree of compatibility with the traditional, visual analysis of EEG.

## Background

Since the first application to EEG in 1995 [1], matching pursuit algorithm (MP) has been shown to significantly improve the EEG/MEG analysis in a variety of paradigms, including pharmaco-EEG [2,3], assessment of propagation [4], dynamics [5] and signal complexity [6-8] in epileptic seizures, detection of seizures [9,10], analysis of somatosensory evoked potentials in humans [11] and rats [12], detection of sleep spindles in Obstructive Sleep Apnea [13] and investigation of their chirping properties [14], studies of high gamma in humans [15] and monkeys [16], investigation of brain’s pain processing [17,18], paramaterization of vibrotactile driving responses [19] and event-related desynchronization and synchronization [20,21].

New area of applications opened with the advent of multivariate MP (MMP) algorithms. MMP preprocessing was shown to significantly improve stability and sensitivity of EEG inverse solutions [22-27] and allowed for tracing evoked responses in single trials of EEG and MEG [28-31].

Finally, the algorithm offers also unique compatibility with the traditional, visual analysis of EEG. Specific mode of operation of MP, which is sequential focusing on locally strongest (“most visible”) signal structures, resembles the working of an electroencephalographer who visually evaluates the EEG time series. Proper interpretation of the MP parameterization can provide a direct link to the results of visual analysis of EEG [32]—that is, we may find a direct correspondence between the waveforms fitted to the EEG time series, and the structures marked by visual scorer, including sleep spindles, slow waves or epileptic spikes [33-35]. This advantage should not be underestimated in the field, where most of our knowledge about behavioral and neurological correlates of EEG comes from visual analysis, which is still the only golden standard and point of reference: according to the Report of the American Academy of Neurology and the American Clinical Neurophysiology Society [36], quantitative EEG analysis *should be used only by physicians highly skilled in clinical EEG, and only as an adjunct to and in conjunction with traditional EEG interpretation*.

In spite of all these advantages, PubMed search of “matching pursuit and EEG” still returns only a two-digit number. This limited acceptance of such a promising method may be partly due to the lack of: 

1. Well defined criteria for setting the most important parameters of the algorithm, which are the number and distribution of dictionary’s functions.

2. Common framework for a variety of multivariate MP algorithms.

3. Robust and user friendly software based upon solid mathematical foundations.

Following sections address these issues from two different angles. Next section *An interactive tour of matching pursuit* provides plain English introduction of the major aspects and parameters of the algorithm, based on example computations. Following sections use equations to introduce optimal sampling of Gabor dictionaries and a common framework for multivariate MP (MMP), and Appendix discusses major numerical and mathematical optimizations used in the MMP implementation accompanying this paper.

## An interactive tour of matching pursuit

**Figure 1:**
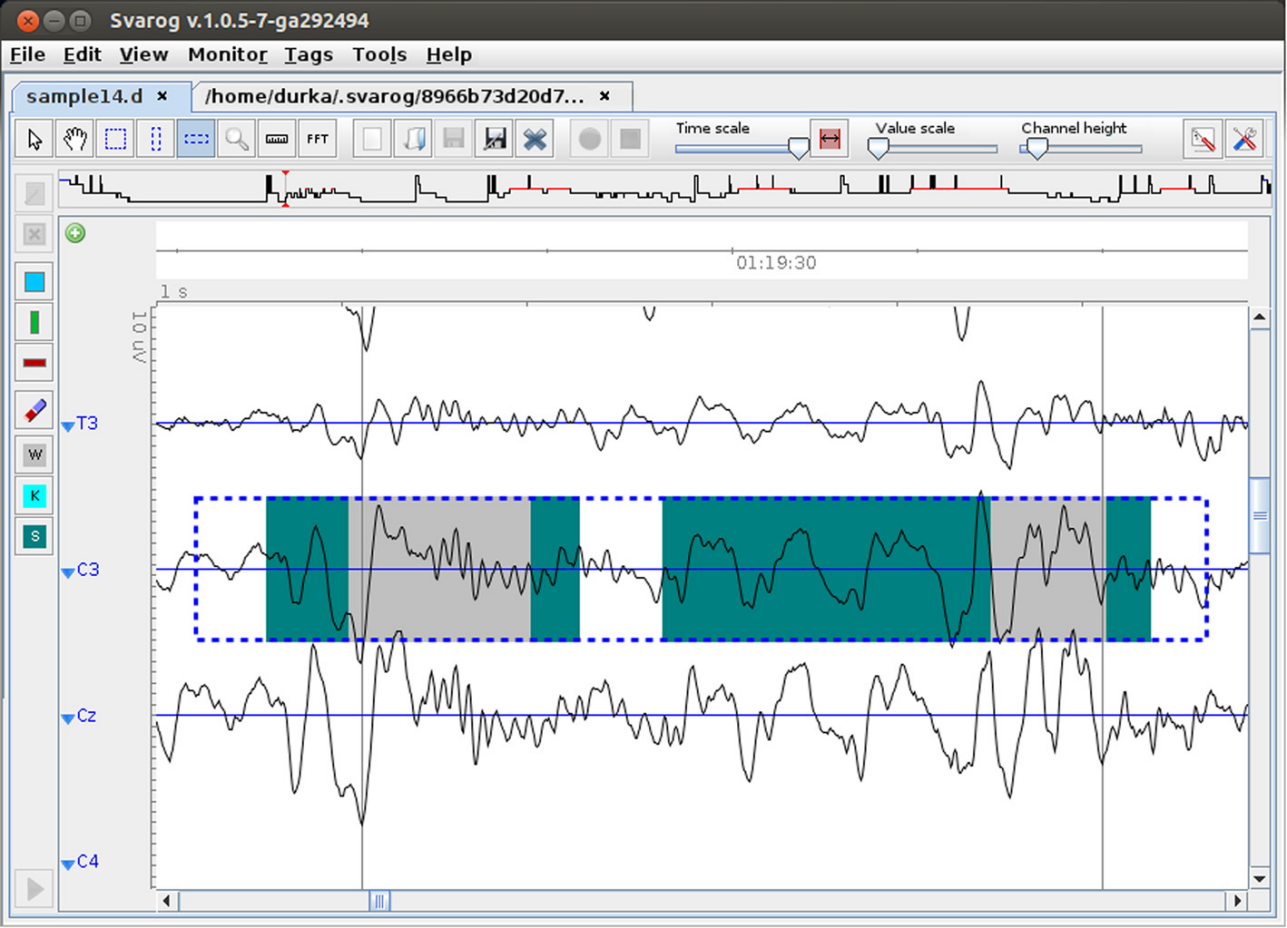
**Sample epoch of sleep EEG.** Screenshot displaying an epoch of sleep EEG recording. SWA and sleep spindles were marked by an electroencephalographer as green and gray rectangles, correspondingly (in this case both spindle tags fall inside the epochs marked as SWA). Blue dashed line outlines the epoch from C3 selected for MP decomposition, shown in Figure 2.

**Figure 2:**
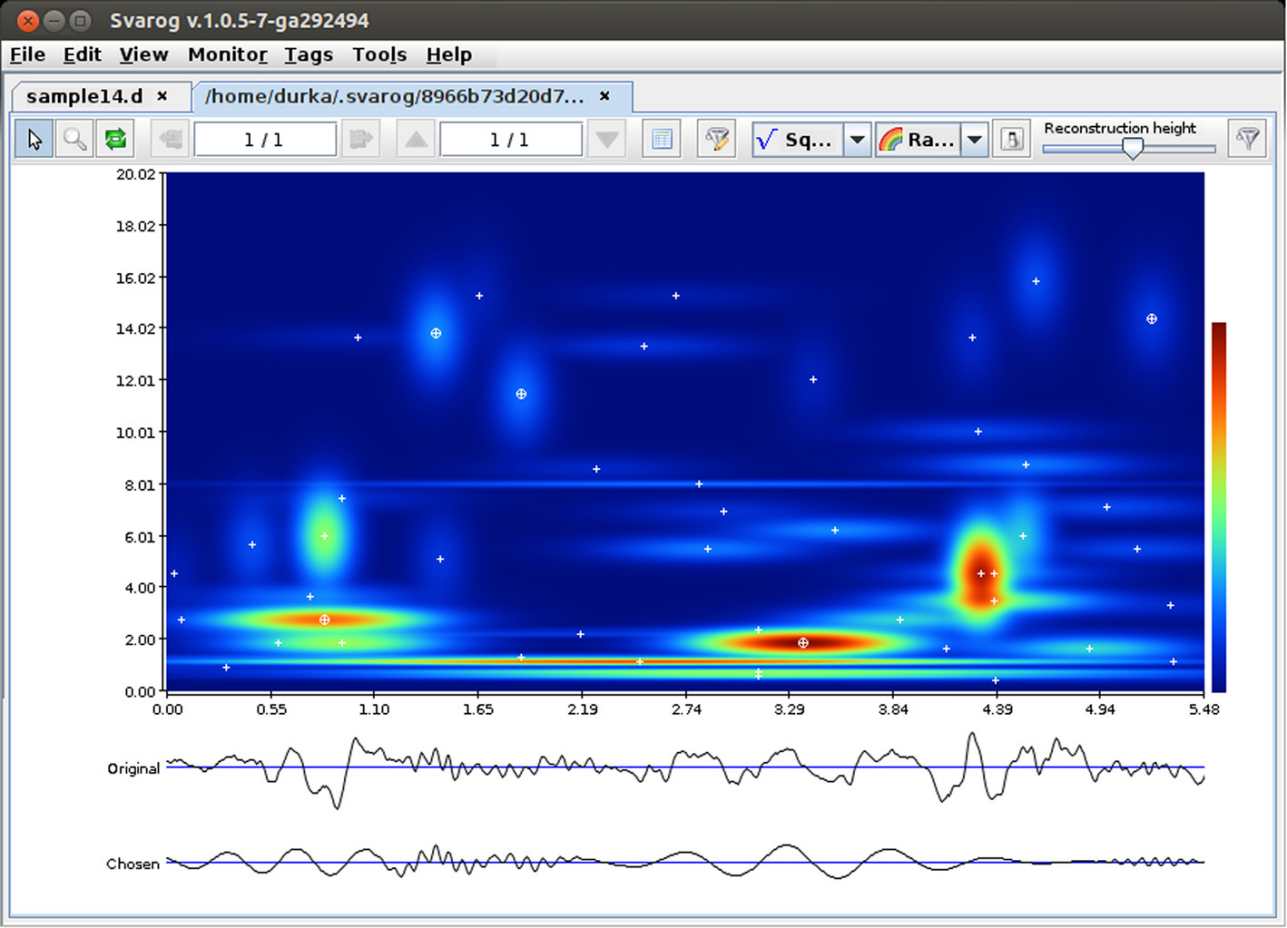
**Time-frequency distribution of signal’s energy.** Results of MP decomposition displayed as an interactive time-frequency of map signal’s energy density in Svarog. Clicking center of a blob (marked by white cross) adds the corresponding function to the reconstruction (bottom signal).

**Figure 3:**
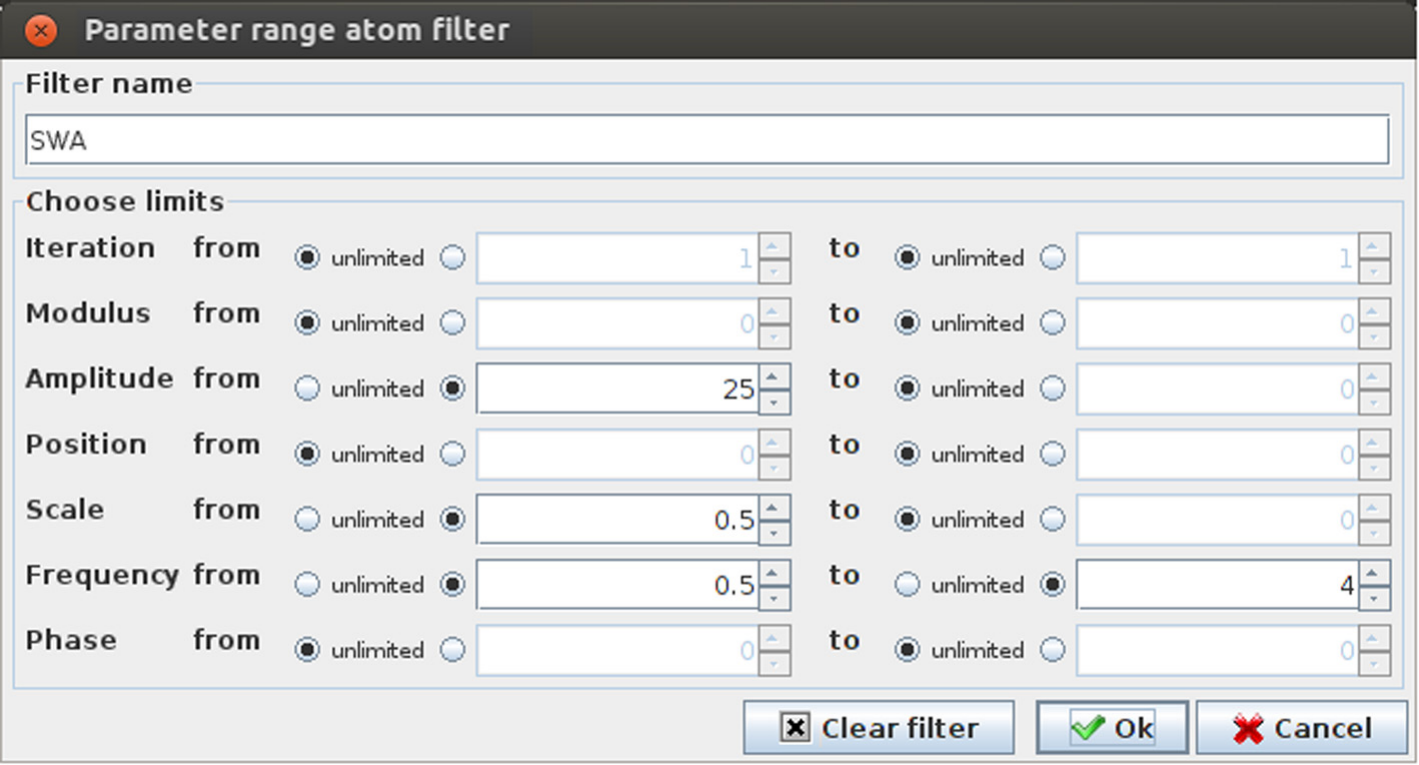
**Defining a filter for SWA.** Filter defining criteria for waveforms corresponding to SWA in the Svarog interface to MP.

**Figure 4:**
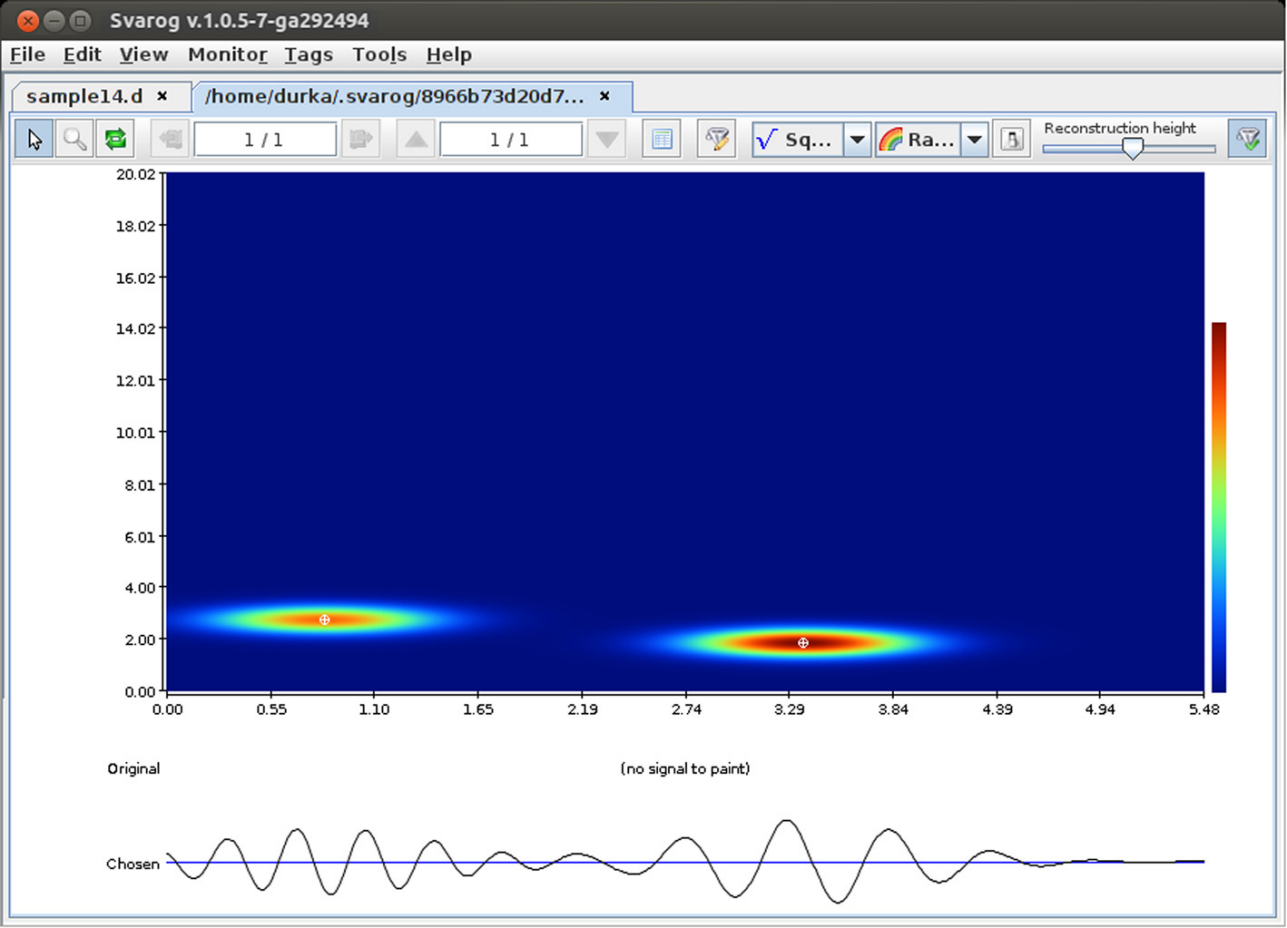
**Structures corresponding to SWA.** Result of the application of the filter from Figure 3 to the decomposition from Figure 2.

**Figure 5:**
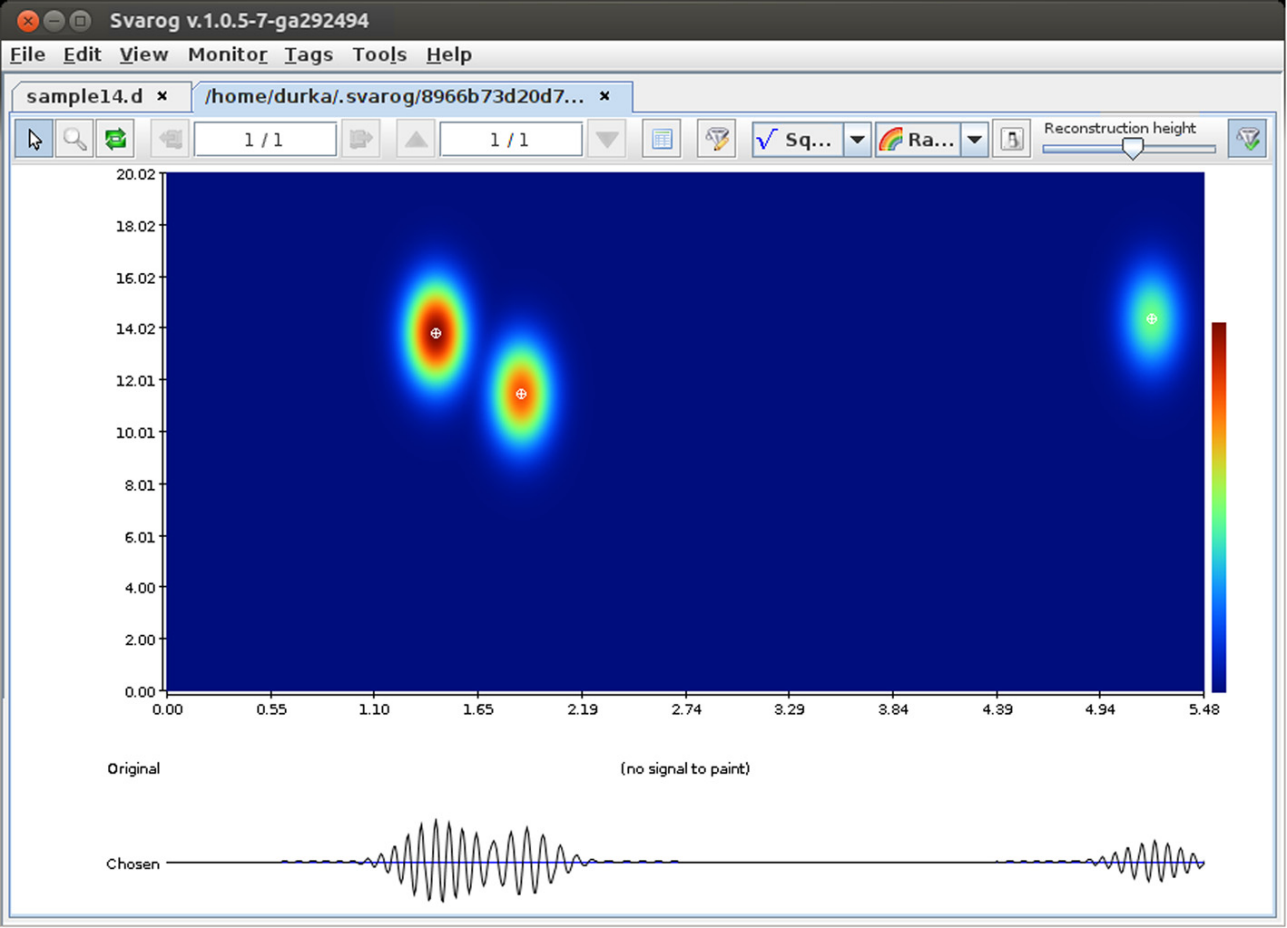
**Structures corresponding to sleep spindles.** Result of application of the filter defining sleep spindles to the decomposition from Figure 2.

**Figure 6:**
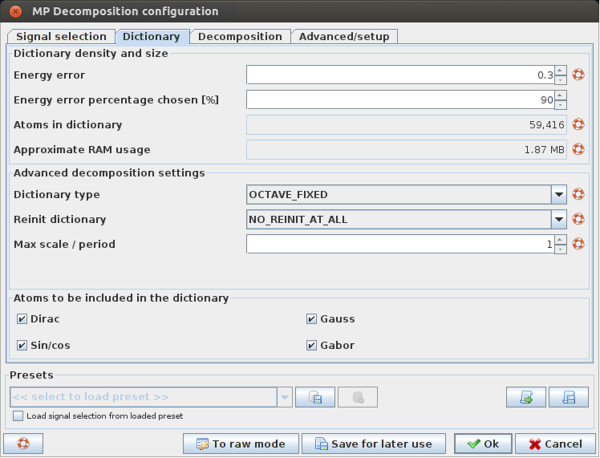
**Setting the dictionary parameters for MP decomposition in Svarog.** Tab for setting the parameters governing construction of the Gabor dictionary for MP decomposition in Svarog.

[Additional file 1] is a video tutorial for downloading and configuration of the Svarog package, used for computations and visualization of results (Figures 1, 2, 3, 4, 5 and 6 are screenshots from Svarog). Screencast in [Additional file 2] shows the steps from loading the signal to displaying interactive map of time-frequency energy distribution. [Additional file 3] contains help of the MP module from Svarog. Complete software environment used for computations presented in this paper (including examples from the following sections) is freely available from http://braintech.pl/svarog, as described in section *Software availability and requirements*.

### The algorithm

The gist of the matching pursuit algorithm can be summarized as follows: 

1. We start by creating a huge, redundant set (called a dictionary) of candidate waveforms for representation of structures possibly occurring in the signal. For the time-frequency analysis of signals we use dictionaries composed of sines with Gaussian envelopes, called Gabor functions, which reasonably represent waxing and waning of oscillations.

2. From this dictionary we choose only those functions, which fit the local signal structures. In such a way, the width of the analysis window is adjusted to the local properties of the signal. Local adaptivity of the procedure is somehow similar to the process of visual analysis, where an expert tends to separate local structure and assess their characteristics. Owing to this local adaptivity, MP is the only signal processing method returning explicit time span of detected structures.

3. The above idea is implemented in an iterative procedure: in each step we find one “best” function, and then subtract it from the signal being decomposed in the following steps.

### MP advantages in EEG

We will discuss some of the advantages of MP in EEG analysis using an example from the field of sleep research. As mentioned in section *Background*, visual analysis of EEG is still the golden standard; in the area of sleep the basic reference [37] comes from 1968 (with later updates [38]). It defines criteria for division of sleep into stages, based mostly upon presence/prevalence of certain structures in the corresponding epochs of EEG recording. As for the definitions of these structures, formulated for standardization of their visual detection, let us take the example of sleep spindles: 

The presence of sleep spindle should not be defined unless it is at least 0.5 sec. duration, i.e. one should be able to count 6 or 7 distinct waves within the half-second period. (...) The term should be used only to describe activity between 12 and 14 cps [37].

Over the years, common definition drifted towards frequencies from 11 to 15 Hz, duration 0.5–2 seconds and amplitude usually above 15 *μV*. Reading this definition after 45 years, we are still surprised that: 

1. Criteria are defined almost explicitly in time-frequency terms.

2. Before 1993 (that is before the introduction of the matching pursuit [39]), no signal processing method returning explicitly the frequency, amplitude and time width of the oscillations present in a signal was known.

In the following we present MP decomposition of an EEG epoch containing sleep spindles and slow wave activity (SWA). Figure 1 presents a sample epoch of sleep EEG loaded into Svarog (Signal Viewer, Analyzer and Recorder on GPL, see section *Software availability and requirements*). Green and gray rectangles represent SWA and sleep spindles, marked visually by an encephalographer.

Figure 2 presents results of MP decomposition of the epoch selected in Figure 1. Curves below the time-frequency map of energy density represent: 

1. Original signal chosen for decomposition (marked in Figure 1).

2. Reconstruction (time course) of selected functions (marked by circled crosses).

Central panel holds the time-frequency map of signals energy density. Each function fitted to the signal by MP is presented as a Gaussian blob in the appropriate time and frequency coordinates, with time and frequency widths corresponding to its parameters. Formula for computing this representation is given by Eq. (4). One can argue about the superior properties of this estimate; indeed, there is a lot of possible ways to estimate signals energy density in the time-frequency plane (spectrogram, wavelets, Wigner-Ville etc.) and none of them is perfect for all signals. Therefore, we shall concentrate on a unique feature of the MP decomposition, which is an explicit parameterization of the transients present in a signal.

Each of the blobs in Figure 2 represents a Gabor function of known time position and width, amplitude, frequency and phase, as in Eq. (3). These parameters contain the primary information about the signal’s content (Eq. (2)), and they can be used directly for the investigation of the properties of the signal, as exemplified below.

Let us define the occurrence of SWA as a wave of amplitude above 50 *μV*, width above half a second and frequency from 0.5 to 4 Hz. Definition of such filter in Svarog is presented in Figure 3. The amplitude is entered as 25 *μV*, because the encephalographic convention relates to the peak-to-peak amplitude, which for the Gabor function is double of the mathematical amplitude. This convention is not the only difference between mathematics and visual perception of structures in the signal. For example, visual perception of both amplitude and time width depends on the context (mostly the variance of the signal). Therefore, if exact replication of the visual detection is the main goal, factors like S/N ratio can be incorporated into the post processing criteria.

Result of the application of the filter from Figure 3 to the decomposition from Figure 2 is given in Figure 4. Automagically, all that is left are structures conforming to the definition of SWA. Two major advantages of this result over previously available methods should be noted: 

1. This selection takes into account all the features defining SWA, not only their frequency content as would be the case e.g. for a bandpass filter.

2. We have separate parameterization of all the conforming structures, including e.g. the duration of each of them.

The latter feature was explored in [40] for detection of sleep stages III and IV. These stages are defined as epochs occupied by SWA in 20–50% and over 50% of their time, correspondingly. As the previously employed signal processing methods did no parametrize explicitly the time span of relevant activity, it was the first explicit implementation of this rule, designed for standardization of visual detection, in a fully automatic system. We observe also a good concordance of these results with visual detection, represented by the two green marks (intersected by the gray marks for spindles) representing visually tagged SWA in Figure 1. This concordance was quantitatively evaluated in [34].

Similar operation can be performed for sleep spindles; application a filter reflecting their time-frequency parameters, quoted at the beginning of this section, gives us Figure 5. Again, structures detected by the algorithm fall within the borders marked by enecephalographer for spindles (gray tags in Figure 1). We observe also a typical example, when two superimposed spindles (on the left) were marked by the human expert as one. It exemplifies the fact that MP-based methods are in most cases downward compatible with visual detection, yet, apart from repeatability and automatization offer also increased sensitivity and resolution. Indeed, in the noisy signal it is almost impossible to see the boundary, and human brain did not evolve for an online calculation of instantaneous frequency, which differentiates these two spindles.

Similarly to the detection of SWA, this scheme is not only more sensitive and selective compared to previous approaches, while retaining a high degree of concordance with visual detection [33,41], but also allows us, for example, to explicitly count the number of spindles occurrences in any given epoch—a parameter also relevant in sleep analysis.

Finally, it is worth noting that the above proposed procedure is essentially free from any method-dependent settings, like the choice of the mother wavelet in wavelet transform, window width in spectrogram, or smoothing kernel in Wigner-Ville derived representations. All these parameters can significantly alter the results of analysis, and optimal settings can be different for each analyzed epoch. On the contrary, matching pursuit decomposition as such is “more or less” uniquely defined by Equation (1). However, subtle relations between dictionaries and results of MP decomposition were not fully understood so far—their explanation constitutes the main mathematical result presented in this paper. So for the rest of this section let us concentrate on the “more or less”.

### Parameters of MP decomposition

As introduced in section MP algorithm, MP searches for functions fitting the signal in a large and redundant set called dictionary. The bigger (more redundant) this dictionary is, the better the chance of a perfect fit. Although it’s a vague statement, nothing substantially more precise had been said on the relation of the dictionary size and quality of MP decomposition—until now.

This study introduces a novel construction of the dictionary, in which the inner products of the adjacent atoms are kept constant. In other words, distribution of dictionary’s functions is uniform with respect to the metric related to their inner product. Using this construction gives us control over the maximum error of a single MP iteration, measured in terms of energy (product of the signal and a function from the optimal dictionary). Upper bound on this error is given by the worst case, where a structure present in the signal falls “in the middle” between dictionary’s functions available for decomposition. This error is not equivalent to the resolution of approximation, because MP is an iterative, nonlinear procedure. Formal introduction of the mentioned metric and construction of the optimal dictionary are given in section *Optimal sampling of Gabor dictionaries*.

Figure 6 presents the “Dictionary” tab from the MP configuration window. The most important parameter (first from the top) is called “Energy error”. It corresponds to *ϵ* from Equation (7) in section *Optimal sampling of Gabor dictionaries*, and relates directly to the mentioned above maximum error of a single MP iteration.

On the practical side, this parameter regulates the price/performance tradeoff in MP decomposition. Smaller (closer to zero) values result in larger amount of functions in the dictionary and higher resolution of resulting MP decomposition, at a price of increased computation times and memory requirements. After each change of this parameter the window displays the amount of RAM that will be occupied by the corresponding dictionary. Keeping “Energy error” constant for analysis of epochs of different sizes will result in larger dictionaries for longer epochs, but the accuracy of decomposition, related to the density of the dictionary, will be the same except for the border effects.

Another issue related to the dictionary is a possible statistical bias, present in decompositions of many epochs in the same, relatively small dictionary. This problem was discussed in [42], where solution in terms of stochastic dictionaries was proposed. Stochastic dictionaries introduced in [42] were constructed by explicit random drawing of functions parameters from defined ranges (subsection *Uniform sampling*). This approach gave less control over the structure of the dictionary and excluded possibilities of several valuable numerical optimizations. In this paper we propose a different approach, where randomization is achieved by random removal of a defined fraction of functions from a dense, structured dictionary. This procedure is applied when user chooses “OCTAVE_STOCH” as “Dictionary type”. In such case, a dictionary is first created according to the parameter ‘Energy error” (*ϵ* from Equation (7)), and then selected fraction of functions is randomly removed from the dictionary. This fraction is governed by the parameter “Percentage chosen”, so that (100−Percentage chosen)*%* of functions is removed.

Finally, the last of the major parameters sets the number of matching pursuit iterations, which is equivalent to the number of functions chosen for the representation (compare Equation (2)). Owing to the convergence property of MP, they are ordered by decreasing energy. Increasing the number of iterations does not influence the parameters of functions chosen in earlier steps.

That is, if we make two separate decompositions of the same signal epoch and using the same dictionary—the first one with 50 iterations and the second one with 10 iterations—then the result of the first decomposition will be the same as taking the first 10 functions from the second decomposition (however, in case of stochastic dictionaries, these two decompositions will be performed using slightly different dictionaries). This difference is more pronounced for smaller dictionaries.

There are several mathematical criteria for stopping the decomposition; their influence was evaluated e.g. for MP-based descriptors of signal complexity [6] or optimal video coding [43]—general discussion of this parameter can be found in [44]. Software presented in this paper implements the two most basic options, based upon the maximum number of iterations (“Max iterations”) and percentage of signal’s energy explained by the whole decomposition (“Energy percent”). These options work in logical conjunction. Hence e.g. for the default settings (99% and 50 iterations), the procedure is completed after the 50th iteration—or before, if the representation (2) explains 99% of energy of the original signal for a lower number of functions *M*.

Detailed information on the parameters of MP decomposition employed in mp5 is available from [Additional file 3], which contains the help of the mp5 module from Svarog.

## Optimal sampling of Gabor dictionaries

We start by formally reintroducing the MP following the notation from the seminal paper by Mallat and Zhang [39]. Denoting the function fitted to the signal *x* in the *n*-th iteration of MP as $g_{\gamma_{n}}$, and *n*-th residual as *R*^*n*^*x*, we define the procedure as:

(1) $$\left\{\begin{array}{l} R^{0}x=x\\ R^{n}x=\langle R^{n}x,g_{\gamma_{n}}\rangle g_{\gamma_{n}}+R^{n+1}x\\ g_{\gamma_{n}}=\arg\;\underset{g_{\gamma_{i}}\in D}{\max}|\langle R^{n}x,g_{\gamma_{i}}\rangle | \end{array}\right)$$

As a result we get an expansion

(2) $$x\approx \overset{M-1}{\underset{n=0}{\sum }}\langle R^{n}x,\;g_{\gamma_{n}}\rangle g_{\gamma_{n}}$$

where *M* equals the number of iterations. For a time-frequency analysis of real-valued signals, dictionary *D* is usually composed from Gabor functions

(3) $$g_{\gamma }(t)=K(\gamma )e^{-\pi {\left(\frac{t-u}{s}\right)}^{2}}\cos\left(\omega (t-u)+\phi \right)$$

where *γ* = (*u*,*ω*,*s*) and *K*(*γ*) is such that ||*g*_*γ*_|| = 1.

From expansion (2) we can derive a time-frequency distribution of the signal’s energy (as shown in Figure 2), by summing Wigner distributions *W* of selected functions and explicitly omitting cross-terms:

(4) $$\text{Ex}(t,\omega )=\overset{M}{\underset{n=0}{\sum }}{\left|\langle R^{n}x,\;g_{\gamma_{n}}\rangle \right|}^{2}\;Wg_{\gamma_{n}}(t,\omega )$$

This magnitude is presented in Figures 2, 4 and 5.

### Real world implementations of Gabor dictionaries

Parameters (*u*,*ω*,*s*) of functions *g*_*γ*_ from Equation (3) form a 3-dimensional continuous subspace of $R^{3}$—the infinite set *D*_*∞*_. Ranges of parameters delimiting this subspace correspond to the usual assumptions that the time center *u* and time spread *s* do not exceed the length of the analyzed epoch, and to the fact that *ω* makes no sense above the Nyquist frequency. The fourth parameter *ϕ* does not have to be taken into account in the construction of the dictionary *D*, since for any *g*_*γ*_ we can find the phase *ϕ* maximizing its product 〈*g*_*γ*_,*x*〉 with given signal *x* in a single step—we recall this method in the Appendix (section *Optimal phase of a Gabor function*).

From this *D*_*∞*_, a finite dictionary *D*⊂*D*_*∞*_ must be chosen for any practical implementation of MP. This choice is equivalent to the choice of a discrete and finite set *γ* from the continuous ranges of *u*, *ω*, and *s*. However, up to now the major questions, crucial for real-world implementations of MP, were unanswered: 

1. How should we choose the elements of the finite dictionary *D* from *D*_*∞*_ and why?

2. How much do we gain by increasing the size of the dictionary?

It has been a well known fact, that using a larger dictionary of functions for decomposition of the same signal should lead to more accurate parametrization. However, no direct relation between the density of the dictionary and resolution of the resulting decomposition was derived so far. While an exact measure of the resolution of the highly nonlinear matching pursuit remains an open question, in the following sections we relate the maximum error of a single MP iteration to the density of an optimally sampled Gabor dictionary, filled with Gabor functions distributed uniformly with respect to the metric related to their inner product.

### Dyadic sampling

The first, wavelet-like subsampling of *D*_*∞*_ was proposed in the seminal paper [39]:

(5) $$\gamma =(u,\omega ,s)=\left(p\;a^{j}\Delta u,\;k\;\frac{\Delta \omega }{a^{j}},\;a^{j}\right)$$

where j,k,p∈Z and the basic intervals for time and frequency (Δ*u* and Δ*ω*) are chosen so that $\Delta u=\frac{\Delta \omega }{2\pi }<1$. If a dictionary *D* is constructed from the functions *g*_*γ*_ with parameters *γ* = (*u*,*ω*,*s*) chosen according to (5), then for any signal $x\in L^{2}(R)$ there exists an optimality factor *α*>0 such that

(6) $$\underset{g_{\gamma }\in D}{\sup}|\langle x,g_{\gamma }\rangle |\geq \alpha \underset{g\in D_{\infty }}{\sup}|\langle x,g\rangle |$$

However, there were no quantitative estimates for the performance of the MP in given dictionary, constructed for given Δ*ω* and *a*, and it seems that nothing can be said about the optimality factor *α* except for that it exists and is greater than zero.

### Uniform sampling

The quest for a justification of a particular scheme of subsampling *D*_*∞*_ leads us to the considerations of a uniform sampling. If we know that the parameters (*u*,*ω*,*s*) are uniformly sampled with steps Δ*u*, Δ*ω*, and Δ*s*, then for any *g*∈*D*_*∞*_ there exists *g*_*γ*_∈*D*, which has the parameters differing at most by halves of the sampling steps. Such sampling provides an estimate of one-step resolution of the highly nonlinear MP algorithm in the space of the parameters of Gabor functions. It was implemented in the mp4 software package [45] as a byproduct of the first approach to the issue of stochastic dictionaries [42], where parameters of the dictionary’s functions were drawn from uniform probability distributions.

However, such a sampling scheme in some cases is far from optimal for the MP algorithm. For example, constant sampling of the time interval Δ*u* for all the widths *s* will result in a dictionary containing a lot of strongly overlapping Gabors with large widths *s* (which will give large inner products with neighbors from the dictionary), and much more sparse coverage of positions of “shorter” Gabors with small *s*. Choice (1) of functions for the MP expansion (2) is based entirely on inner products, and the Cartesian metric in the space of the parameters of these functions is far from optimal in this respect. This leads us to the search for a metric that would correspond to the MP selection procedure (1).

### In pursuit of a relevant metric

A metric that would reflect the distance between the dictionary elements as “seen” by the MP algorithm should be based on the inner product of dictionary’s elements. We propose a metric *d*(*g*_0_,*g*_1_) to ensure that the distance between nearest Gabor atoms in the dictionary does not exceed some *a priori* given threshold, *ϵ*. Similarly to dyadic dictionary (see Eq. 5), we assume that the scale parameter *s* varies by a factor *a* (dilation factor), as follows: *s*_*j*_=*a*^*j*^, where *j* > 0 and *a* > 1. However, we will propose a new method for determination of step values Δ*ω* and Δ*u*. We will show that this parametrization allows us to derive such step values, that for every (*s*,*ω*,*u*)

(7) $$\begin{array}{rr} d(g_{\left(s,\omega ,u,\phi \right)},g_{\left(\text{as},\omega ,u,\phi \right)}) & \leq \epsilon \\ d(g_{\left(s,\omega ,u,\phi \right)},g_{\left(s,\omega +\Delta \omega ,u,\phi \right)}) & \leq \epsilon \\ d(g_{\left(s,\omega ,u,\phi \right)},g_{\left(s,\omega ,u+\Delta u,\phi \right)}) & \leq \mathrm{\epsilon .} \end{array}$$

In order to construct a proper metric *d*(*g*_0_,*g*_1_), we start by introducing a simple, intuitive measure for Gabor function similarity which satisfies all the properties of a metric:

(8) $$d_{0}(g_{0},g_{1})=2^{-\frac{1}{2}}\left\|g_{0}-g_{1}\right\|=2^{-\frac{1}{2}}\sqrt{\langle g_{0}-g_{1}|g_{0}-g_{1}\rangle }=\sqrt{1-\langle g_{0}|g_{1}\rangle }.$$

Norm *d*_0_ above is naturally defined based upon the inner product in the space of the real signals. The constant $2^{-\frac{1}{2}}$ has been introduced to set the distance between orthogonal Gabor atoms to 1, while greater distances (up to 2) can exist between Gabor atoms whose inner products are negative.

Inner product of two Gabor functions has the dimension of energy, thus the dimension of the metric (8) is amplitude. According to this fact, the conditions (7) imply the dimension of the square of the parameter *ϵ* as the quantity of energy. Also, 〈 *G*_0_ | *G*_1_〉_max_ will be nonnegative for any two Gabor functions. Therefore, since Gabors are normalized, the value of their inner product is limited to the range (0,1) and thus the limit of the *ϵ* is (0,1). However, *ϵ* = 1 allows the neighboring atoms to be orthogonal, so for a reasonable MP decomposition one should require *ϵ* ≪ 1.

To the best of our knowledge, the above measure has not been previously applied to the Gabor dictionary construction problem, although a similar one for packing in projection spaces was introduced by Tropp [46]. Interpretation and possible values of parameter *ϵ* will be discussed in the following section, after we construct a final metric based on *d*_0_(*g*_0_,*g*_1_).

### Phase-related equivalence

According to the section *Optimal phase of a Gabor function* from the Appendix, one can easily calculate phase *ϕ* of the Gabor function that maximizes its inner product with a given signal. Therefore, each atom in MP dictionary can be replaced with any other representative from the set *G*_(*s*,*ω*,*u*)_, which is an equivalence class in respect to a relation ∼ defined as

(9) $$g_{\left(s_{0},\omega_{0},u_{0},\phi_{0}\right)}∼g_{\left(s_{1},\omega_{1},u_{1},\phi_{1}\right)}\;\Leftrightarrow \;(s_{0}=s_{1})\wedge (\omega_{0}=\omega_{1})\wedge (u_{0}=u_{1}).$$

This feature has to be taken into account by introducing a correct metric for Gabor atoms

(10) $$d(g_{0},g_{1})=\tilde{d}(g_{0}/\;∼,g_{1}/\;∼),$$

where $\tilde{d}(G_{0},G_{1})$ is the distance function defined on equivalence classes

(11) $$\tilde{d}(G_{0},G_{1})=\underset{\begin{array}{c} g_{0}\in G_{0}\\ g_{1}\in G_{1} \end{array}}{\min}d_{0}(g_{0},g_{1})=\sqrt{1-\underset{\begin{array}{c} g_{0}\in G_{0}\\ g_{1}\in G_{1} \end{array}}{\max}\langle g_{0}|g_{1}\rangle }=\sqrt{1-{\langle G_{0}|G_{1}\rangle }_{\text{max}}},$$

and we introduce the *maximal scalar product* 〈 *G*_0_ | *G*_1_ 〉_max_, which can be calculated as

(12) $${\langle G_{\left(s_{0},\omega_{0},u_{0}\right)}|G_{\left(s_{1},\omega_{1},u_{1}\right)})\rangle }_{\text{max}}=\underset{\phi_{0},\phi_{1}\in [0;2\pi ]}{\max}\langle g_{\left(s_{0},\omega_{0},u_{0},\phi_{0}\right)}|g_{\left(s_{1},\omega_{1},u_{1},\phi_{1}\right)}\rangle .$$

### Inner products of Gabor functions

The measure *d*_0_ (8) is based on an inner product of Gabor functions, so it is necessary to calculate analytical formulae for this product. Such expressions can be found for real Gabor functions

(13) $$g_{\gamma }(t)=K_{\gamma }e^{-\pi {\left(\frac{t-u}{s}\right)}^{2}}\cos(\omega (t-u)+\phi ),$$

where *γ* ≡ (*s*,*ω*,*u*,*ϕ*) is a set of the parameters, and

(14) $$K_{\gamma }=\frac{2^{3/4}}{\sqrt{s}}{\left[1+\cos(2\phi )e^{-\frac{s^{2}\omega^{2}}{2\pi }}\right]}^{-\frac{1}{2}}$$

is the normalization factor.

To present an analytical formula for the inner product of two real Gabor functions (13) *g*_0_ and *g*_1_ with normalization factors *K*_0_ and *K*_1_ respectively, we introduce following constants:

(15) $$A=\pi \left(\frac{1}{s_{0}^{2}}+\frac{1}{s_{1}^{2}}\right)$$

(16) $$B=\frac{\pi }{A}\left(\frac{u_{0}}{s_{0}^{2}}+\frac{u_{1}}{s_{1}^{2}}\right)$$

(17) $$C=-\pi \left(\frac{u_{0}^{2}}{s_{0}^{2}}+\frac{u_{1}^{2}}{s_{1}^{2}}\right)+AB^{2}=-\pi \frac{{\left(u_{0}-u_{1}\right)}^{2}}{s_{0}^{2}+s_{1}^{2}}.$$

The complete formula for the product has been adapted from [47]:

(18) $$\begin{array}{ll} \langle g_{0},g_{1}\rangle & =K_{0}K_{1}\sqrt{\frac{\pi }{4A}}e^{C}\left(\cos\left[\left(\omega_{0}+\omega_{1})B+(\phi_{0}+\phi_{1})-(\omega_{0}u_{0}+\omega_{1}u_{1}\right)\right]e^{-\frac{{\left(\omega_{0}+\omega_{1}\right)}^{2}}{4A}}+\right)\\ & +\left(\cos\left[\left(\omega_{0}-\omega_{1})B+(\phi_{0}-\phi_{1})-(\omega_{0}u_{0}-\omega_{1}u_{1}\right)\right]e^{-\frac{{\left(\omega_{0}-\omega_{1}\right)}^{2}}{4A}}\right). \end{array}$$

### Products of adjacent atoms

To simplify the inner product (18) for special cases of adjacent dictionary atoms, we will calculate distances between two atoms which differ in only one of the three parameters *s*,*ω*,*u*. Let us discuss each case separately.

#### Scale

Because *u*_0_=*u*_1_, the scalar product is invariant to a shift in position *u*, so we can safely choose *u*=0. In this case $A=\frac{\pi (a^{2}+1)}{a^{2}s^{2}}$, *B*=0, *C*=0. Therefore,

(19) $$\langle g_{\left(s,\omega ,u=0,\phi_{0}\right)}|g_{\left(\text{as},\omega ,u=0,\phi_{1}\right)}\rangle =\sqrt{\frac{2a}{a^{2}+1}}\frac{\cos(\phi_{0}-\phi_{1})+\cos(\phi_{0}+\phi_{1})e^{-\frac{a^{2}s^{2}\omega^{2}}{(a^{2}+1)\pi }}}{\sqrt{1+\cos(2\phi_{0})e^{-\frac{s^{2}\omega^{2}}{2\pi }}}\sqrt{1+\cos(2\phi_{1})e^{-\frac{a^{2}s^{2}\omega^{2}}{2\pi }}}}.$$

The global maximum of this product will be present for *ϕ*_0_=*ϕ*_1_=0 or $\phi_{0}=\phi_{1}=\frac{\pi }{2}$, depending on parameter values. Therefore, one can calculate

(20) $$\;{\langle G_{\left(s,\omega ,u\right)}\;|G_{\left(\text{as},\omega ,u\right)}\rangle }_{\text{max}}\;=\;\sqrt{\;\frac{2a}{a^{2}+1}}\;\max\;\;\left\{\;\;\frac{1\;-\;e^{-\frac{a^{2}s^{2}\omega^{2}}{(a^{2}+1)\pi }}}{\sqrt{1\;-\;e^{-\frac{s^{2}\omega^{2}}{2\pi }}}\;\sqrt{1\;-\;e^{-\frac{a^{2}s^{2}\omega^{2}}{2\pi }}}};\;\frac{1\;+\;e^{-\frac{a^{2}s^{2}\omega^{2}}{(a^{2}+1)\pi }}}{\sqrt{1\;+\;e^{-\frac{s^{2}\omega^{2}}{2\pi }}}\;\sqrt{1\;+\;e^{-\frac{a^{2}s^{2}\omega^{2}}{2\pi }}}}\;\right\}\;\;.$$

#### Frequency

The frequency parameter *ω* changes by Δ*ω*, *s*_0_=*s*_1_ and *u*_0_=*u*_1_. We can choose *u*=0, analogically to the previous case. Here $A=\frac{2\pi }{s^{2}}$, *B*=0 and *C*=0, so the product is as follows:

(21) $$\;\langle \;g_{\left(s,\omega ,u=0,\phi_{0}\right)}|g_{\left(s,\omega +\Delta \omega ,u=0,\phi_{1}\right)}\rangle =e^{-\frac{\Delta \omega^{2}s^{2}}{8\pi }}\frac{\cos(\phi_{0}-\phi_{1})+\cos(\phi_{0}+\phi_{1})e^{-\frac{s^{2}\omega (\omega +\Delta \omega )}{2\pi }}}{\sqrt{1+\cos(2\phi_{0})e^{-\frac{s^{2}\omega^{2}}{2\pi }}}\sqrt{1+\cos(2\phi_{1})e^{-\frac{s^{2}{\left(\omega +\Delta \omega \right)}^{2}}{2\pi }}}}.$$

The global maximum of this product will be present for $\phi_{0}=\phi_{1}=\frac{\pi }{2}$. Therefore,

(22) $${\langle G_{\left(s,\omega ,u\right)}|G_{\left(s,\omega +\Delta \omega ,u\right)}\rangle }_{\text{max}}=e^{-\frac{\Delta \omega^{2}s^{2}}{8\pi }}\frac{1-e^{-\frac{s^{2}\omega (\omega +\Delta \omega )}{2\pi }}}{\sqrt{1-e^{-\frac{s^{2}\omega^{2}}{2\pi }}}\sqrt{1-e^{-\frac{s^{2}{\left(\omega +\Delta \omega \right)}^{2}}{2\pi }}}}.$$

#### Position

In this case, *s*_0_=*s*_1_, *ω*_0_=*ω*_1_, and position parameter *u* differs by Δ*u*. We can choose $u_{0}=-\frac{\Delta u}{2}$ and $u_{1}=\frac{\Delta u}{2}$. Applying $A=\frac{2\pi }{s^{2}}$, *B*=0, $C=-\frac{\pi \Delta u^{2}}{2s^{2}}$ lead to

(23) $$\;\langle \;g_{\left(s,\omega ,u_{0}=-\frac{\Delta u}{2},\phi_{0}\right)}|g_{\left(s,\omega ,u_{1}=\frac{\Delta u}{2},\phi_{1}\right)}\rangle =e^{-\frac{\pi \Delta u^{2}}{2s^{2}}}\frac{\cos(\phi_{0}-\phi_{1}+\mathrm{\omega \Delta u})+\cos(\phi_{0}+\phi_{1})e^{-\frac{s^{2}\omega^{2}}{2\pi }}}{\sqrt{1+\cos(2\phi_{0})e^{-\frac{s^{2}\omega^{2}}{2\pi }}}\sqrt{1+\cos(2\phi_{1})e^{-\frac{s^{2}\omega^{2}}{2\pi }}}}.$$

The global maximum of the product will appear for *ϕ*_0_=−*ϕ*_1_. Therefore,

(24) $${\langle G_{\left(s,\omega ,u\right)}|G_{\left(s,\omega ,u+\Delta u\right)}\rangle }_{\text{max}}=e^{-\frac{\pi \Delta u^{2}}{2s^{2}}}\underset{\phi \in [0;2\pi ]}{\max}\frac{\cos(2\phi +\mathrm{\omega \Delta u})+e^{-\frac{s^{2}\omega^{2}}{2\pi }}}{1+\cos(2\phi )\;e^{-\frac{s^{2}\omega^{2}}{2\pi }}}.$$

### Construction of the optimally sampled dictionary

Formulae (20), (22) and (24) substituted into (7) could be used to construct an optimal dictionary. However, to improve computational performance of MP algorithms, one can use Fast Fourier Transform (FFT) as described in the Appendix. Therefore, it would be preferable to have values of Δ*ω* and Δ*u* independent of *ω*, yet still satisfying (7).

**Figure 7:**
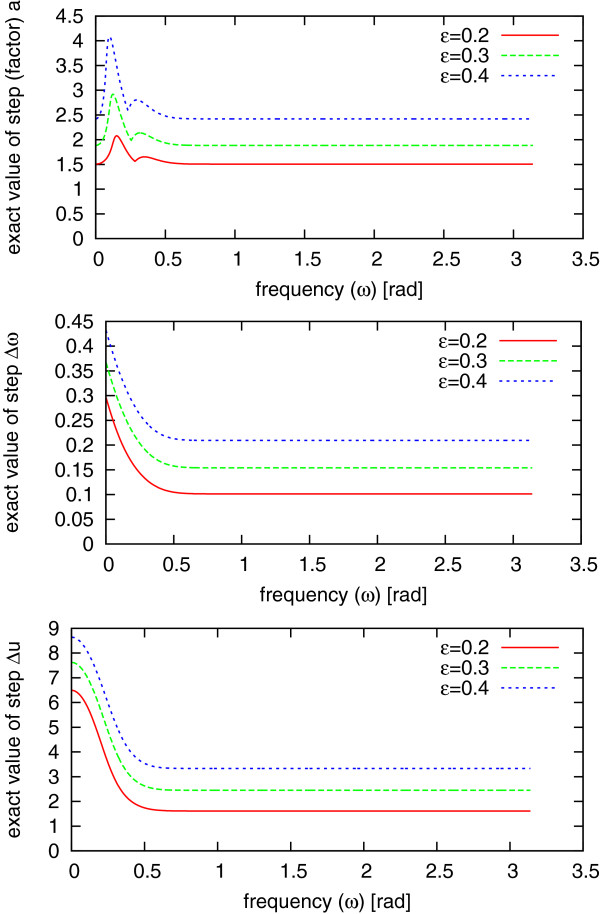
**Optimal scale factor, frequency step and position step.** Optimal scale factor *a* (top plot—see Eq. (20)), frequency step Δ*ω* (middle plot—see Eq. (22)) and position step Δ*u* (see Eq. (24)) as a function of *ω* for three values of *ϵ* and scale *s*=10.

To construct such “uniform” dictionary, step values (*a*, Δ*ω*, Δ*u*) must be selected for such *ω* that minimizes the value of the maximal scalar product (12). Exemplary step values obtained from (20), (22) and (24) are shown in Figure 7, as a function of frequency *ω*, for three different values of parameter *ϵ*. It can be observed that in order to obtain *a*, Δ*ω*, Δ*u* fulfilling the condition (7) and independent of *ω*, one has to select step values corresponding to a large value of frequency *ω*.

Moreover, at frequency *ω*≈*π*, one can safely substitute in Equations (20), (22) and (24):

(25) $$\begin{array}{rr} e^{-\frac{s^{2}\omega^{2}}{2\pi }} & \approx 0\\ e^{-\frac{a^{2}s^{2}\omega^{2}}{2\pi }} & \approx 0\\ e^{-\frac{a^{2}s^{2}\omega^{2}}{(a^{2}+1)\pi }} & \approx 0\\ e^{-\frac{s^{2}\omega {\left(\omega +\Delta \omega \right)}^{2}}{2\pi }} & \approx 0. \end{array}$$

With these approximations, maximal scalar products can be calculated easily as

(26) $$\begin{array}{rr} {\langle G_{\left(s,\omega ,u\right)}|G_{\left(\text{as},\omega ,u\right)}\rangle }_{\text{max}} & \approx \sqrt{\frac{2a}{a^{2}+1}}\\ {\langle G_{\left(s,\omega ,u\right)}|G_{\left(s,\omega +\Delta \omega ,u\right)}\rangle }_{\text{max}} & \approx e^{-\frac{\Delta \omega^{2}s^{2}}{8\pi }}\\ {\langle G_{\left(s,\omega ,u\right)}|G_{\left(s,\omega ,u+\Delta u\right)}\rangle }_{\text{max}} & \approx e^{-\frac{\pi \Delta u^{2}}{2s^{2}}}, \end{array}$$

taking into account that max*ϕ*∈[0;2*π*] cos(2*ϕ*+*ω*Δ*u*)=1.

Therefore, the optimal step values independent of *ω* can be calculated from Equations (7) in the following steps: 

1. For a given threshold *ϵ* (see Eq. 7) one can calculate the dilation factor *a*:

(27) $$a=\frac{1+\epsilon \sqrt{\left(2-\epsilon^{2})(\epsilon^{4}-2\epsilon^{2}+2\right)}}{{\left(1-\epsilon^{2}\right)}^{2}}$$

2. The set of scales $s_{1},s_{2},\ldots s_{N_{s}}$ of Gabors are determined as:

(28) $$s_{j}=a^{j},$$

where $j\in Z_{+}$, *N*_*s*_ = ⌊log*aN*⌋ is the number of different scales and *N* is the length of the analyzed signal.

3. For each scale *s* obtained from Eq. (28) the step Δ*ω* in frequency domain and Δ*u* in time domain is calculated according to the formula:

(29) $$\begin{array}{ll} \Delta \omega & =\frac{1}{s}\sqrt{-8\pi \log(1-\epsilon^{2})}\; \end{array}$$

(30) $$\begin{array}{ll} \Delta u & =s\sqrt{-\frac{2}{\pi }\log(1-\epsilon^{2})}.\; \end{array}$$

## Multivariate matching pursuit (MMP)

Need to analyze jointly more than one epoch usually arises in case of simultaneous measurements of more than one signal, or when subsequent epochs are treated as realizations of the same process (usually via some kind of ergodicity assumption). In EEG/MEG analysis these two cases usually refer to: 

1. Simultaneous recording of EEG/MEG from more than one electrode/sensor, called hereafter “channels”.

2. EEG/MEG recording of subsequent time-locked responses to repetitive stimuli (event-related potentials or fields, ERPs/ERFs), called hereafter “trials”.

Several versions of the MMP algorithm were separately proposed for particular applications—we will briefly discuss this issue in section *Discussion*. In this paper we propose a general framework, where variants of the MMP are identified on the basis of mathematical formulation of the two crucial elements of the multivariate algorithm: 

A. Inter-channel/inter-trial constrains, that is parameters that we keep constant or allow to change across the channels/trials.

B. Criteria for selection of the function from the dictionary in each MMP iteration—in MMP, contrary to MP, this function is fitted to many epochs at the same time, and optimality of fit to many signals at once can be expressed in different ways.

Such an universal approach allows for a direct implementation of the same code to a multitude of different paradigms encountered in recordings of any multivariate time series, not only EEG/MEG. Nevertheless, through this paper we will stick to “channels” and “epochs” in relation to the organization of the multivariate datasets. The following chapters define basic variants of MMP.

### MMP1 (max sum of the moduli of products, constant phase)

The most straightforward multivariate extension of the MP—let’s call it MMP1—can be achieved as follows: 

A1. Only the amplitude varies across channels.

B1. We maximize the sum of products in all the channels.

We maximize the sum of moduli of the products rather than their squares, as proposed in [48], due to the more efficient selection of the common phase for all the channels. Also, owing to the Gabor update formula (38), in all the iterations but the first one we compute the products of dictionary’s atoms with the function from the previous iteration, which has all the parameters fixed except for the amplitude. This saves a lot of computations comparing to the case of computing products with all the channels residua separately, as in MMP3.

Let us denote the multichannel signal as **x**, and the signal in the *i*th channel as **x**^*i*^, with $i=1\ldots N_{c}$, where *N*_*c*_ is the number of channels. We may express the condition (B1) for the choice of atom *g*_*γ*_ in *n*-th iteration as

(31) $$\underset{g_{\gamma }\in D}{\max}\overset{N_{c}}{\underset{i=1}{\sum }}|\langle R^{n}x^{i},g_{\gamma }\rangle |$$

The whole procedure can be described as

(32) $$\left\{\begin{array}{l} R^{0}x=x\\ R^{n}x^{i}=\langle R^{n}x^{i},g_{\gamma_{n}}\rangle g_{\gamma_{n}}+R^{n+1}x^{i}\\ g_{\gamma_{n}}=\arg\;\underset{g_{\gamma }\in D}{\max}\sum_{i=1}^{N_{c}}|\langle R^{n}x^{i},g_{\gamma }\rangle | \end{array}\right)$$

Results of MMP1 are given in terms of functions $g_{\gamma_{n}}$, selected in consecutive iterations, and their weights in all the channels, determined for channel *i* by the real-valued products $\langle R^{n}x^{i},g_{\gamma_{n}}\rangle$. In each iteration, the multichannel residuum *R*^*n*+1^**x** is computed by subtracting from the previous residua in each channel *i* the contribution of $g_{\gamma_{n}}$, weighted by $\langle R^{n}x^{i},g_{\gamma_{n}}\rangle$.

### MMP2 (max modulus of the sum of products, constant phase)

Assumption of invariant phase in all the channels was explored in [22] to yield an efficient decomposition algorithm. If we modify the criterion of choice from the previous section to

(33) $$\underset{g_{\gamma }\in D}{\max}\left|\overset{N_{c}}{\underset{i=1}{\sum }}\langle R^{n}x^{i},g_{\gamma }\rangle \right|,$$

we get the conditions: 

A2. Only the amplitude varies across the channels.

B2. We maximize the absolute value of the sum of products across channels.

Due to the linearity of the residuum operator *R*[22], this choice allows for implementing a simple trick. Instead of finding in each step the product of each dictionary’s waveform with all the channels separately, and then computing their sum (33), in each step we decompose the average signal $\bar{x}$

(34) $$\bar{x}=\frac{1}{N_{c}}\overset{N_{c}}{\underset{i=1}{\sum }}x^{i}$$

(35) $$\left\{\begin{array}{l} R^{0}\bar{x}=\bar{x}\\ R^{n}\bar{x}=\langle R^{n}\bar{x},g_{\gamma_{n}}\rangle g_{\gamma_{n}}+R^{n+1}\bar{x}\\ g_{\gamma_{n}}=\arg\;\max_{g_{\gamma }\in D}|\langle R^{n}\bar{x},g_{\gamma }\rangle |\\ R^{n}x^{i}=\langle R^{n}x^{i},g_{\gamma_{n}}\rangle g_{\gamma_{n}}+R^{n+1}x^{i} \end{array}\right)$$

This procedure yields a computational complexity close to the monochannel MP—compared to MMP1, reduced by the factor *N*_*c*_ (that is, number of channels).

Convergence of this procedure may be relatively slower for waveforms appearing in different channels with exactly opposite phases.

Due to operating on the average of channels, this version of the algorithm cannot be directly applied to the data presented in the average reference (montage). These problems are absent in MMP1 as well as in the next implementation, allowing for arbitrary phases across the channels.

### MMP3 (variable phase)

A3. Phase and amplitude vary across the channels.

B3. We maximize the sum of squared products (energies) across channels.

Again, as in (31), we maximize

(36) $$\underset{g_{\gamma }\in D}{\max}\overset{N_{c}}{\underset{i=1}{\sum }}|\langle R^{n}x^{i},g_{\gamma }^{i}\rangle {|}^{2}$$

but this time $g_{\gamma_{n}}^{i}$ are not the same $g_{\gamma_{n}}$ for all channels *i*—they can have different phases.

(37) $$\{\begin{array}{l} R^{0}x=x\\ R^{n}x^{i}=\langle R^{n}x^{i},g_{\gamma_{n}}^{i}\rangle g_{\gamma_{n}}^{i}+R^{n+1}x^{i}\\ g_{\gamma_{n}}=\arg\;\max_{g_{\gamma }\in D}\overset{N_{c}}{\underset{i=1}{\sum }}|\langle R^{n}x^{i},\overset{i}{\underset{\gamma }{g}}\rangle {|}^{2} \end{array}$$

As presented in the Appendix (section *Optimal phase of a Gabor function*), computing an optimal phase of Gabor function *g*_*γ*_, maximizing absolute value of the product 〈*R*^*n*^**x**^*i*^,*g*_*γ*_〉, can be implemented very efficiently. Value of (31) for phases optimized separately will never be smaller than in the case of the phase common to all the channels, so this freedom should improve the convergence.

### MMPXY

In case of multichannel recordings of event-related potentials (ERPs), we are dealing with a slightly more complicated structure of the data. Instead of a vector of signals **x**^*i*^, where *i* indexes channels/sensors, we get a matrix of signals $X_{k}^{i}$, with additional index *k* reflecting subsequent repetitions (single trials). On such data, MMP can be performed across either of the two dimensions *i* and *k*. For the sake of simplicity, we will call these indices “channels” and “trials”, although for the algorithm they can represent any direction of a multidimensional dataset. The algorithm does not contain any problem-specific optimizations and as such preserves generality.

Usually, the first index in multiplexed multichannel data is the channel (sensor) *i* and the second is the time index *t*. Number of trial *k* comes next, and indexes the the set of whole epochs and all channels *i*, related to the *k*-th repetitions of an event (usually ERP). MP operates on epochs indexed by discrete time *t*. MMP will operate on channel and trial indices *i* and *k*, allowing for different constrains across sensors or repetitions.

For the mp5 package we assumed the naming convention in the form MMPXY, where X denotes the version of MMP algorithm used in each iteration across the channel dimension, and Y—across the repetitions: 

**MMP11:** For each channel and trial fit the optimal *g*_*γ*_ with *ϕ*=const.

**MMP12:** Average trials *k* in each channel *i* and fit optimal *g*_*γ*_ with *ϕ*=const to these averages.

**MMP21:** For each trial *k* find the average across channels *i* and fit optimal *g*_*γ*_ with *ϕ*=const to these averages.

**MMP23:** For each trial *k* find the average across channels *i* and fit to these averages optimal *g*_*γ*_ with potentially different phase *ϕ* in each channel.

**MMP32:** For each channel *i* find the average across trials *k* and fit to these averages optimal *g*_*γ*_ with potentially different phase *ϕ* in each trial.

**MMP33:** For each channel and trial fit the optimal *g*_*γ*_ with potentially different phase *ϕ* in each channel and trial.

For example, MMP12 denotes a setting where in each iteration the choice of the atom fitting best all the channels will be effectuated according to MMP1, while across the trials MMP2 will be used. That is, in each iteration the residua of single trials are averaged separately in each channel, and to these averages the best *g*_*γ*_ is fitted according to MMP1. Naming of the algorithms (MMPX) corresponds to the three above subsections. MMP13 and MMP31 were not implemented in mp5.

## Discussion

### Resolution of MP

One of the problems, faced by everyone applying MP to exploratory analysis of signals, is “how big should be the dictionary so that I do not miss some important structures?” Of course “the bigger the better”, but increasing the size of the dictionary increases significantly the computational cost, and the exact gain in resolution of MP representation was not known so far. Optimal Gabor dictionaries, introduced in this paper, for the first time allow to relate the density of the dictionary to the maximum error of a single iteration of the algorithm.

### Details of implementation

This paper describes mathematical foundations and numerical optimizations, implemented in the freely available software package mp5, developed at the University of Warsaw for decomposition of signals using multivariate matching pursuit. This software made possible several published works [24,28,29] plus some studies in progress. It builds on another decade of experience in using the previous, monovariate version of the algorithm which we made freely available as mp4 at http://eeg.pl/mp, and used in over a dozen published studies. Although all these cited works would not be possible without this software, they were published as “classical scientific papers” where author is supposed to concentrate only on the scientific novelty, not tools. There was no space for all the important details of the implementations, which make a big difference in the results and hence constitute the core of the Reproducible Research. Therefore, although the source code of both these packages have been freely available for years, a complete description of employed mathematical and numerical optimizations and tricks, as presented in Appendix 1, was not published up to now.

### Applications of MMP to EEG/MEG

Probably the most promising field of future applications is related to the multivariate matching pursuit (MMP). Real world applications were made possible owing to the progress in computer hardware in recent decades. In section *Multivariate matching pursuit (MMP)* we propose, in concordance with [44], a simple classification of MMP variants according to the combinations of the constraints on parameters and criteria of choice. However, there is a potential continuum of other approaches outside of this simple scheme, tailored very specifically for particular applications. Most of them suffer from the need of arbitrary setting some parameter that may significantly change the results of decomposition. In this light we believe that in most cases the simple approach proposed in section *Multivariate matching pursuit (MMP)*, which is free from arbitrary parameters, is optimal and most elegant, at least for starting. A procedure that is free of task-specific settings has also obvious advantages stemming directly from its generality.

However, specific approaches of course may offer addition advantages in particular cases. For example, MMP tailored for the analysis of stereo recordings of sound in [49] allows for different time positions of the time-frequency atoms present in the two channels. Together with different amplitudes in each channel, it relates to modeling the microphones as gain-delay filters in the anechoic case. Unfortunately, a model explaining relations between channels of EEG/MEG recordings is far more complicated, even in the case of known distribution of sources (so-called forward EEG problem).

An attempt to incorporate constraints, reflecting the generation of multichannel EEG, into the MMP procedure, was presented in [26]. To the purely energetic criterion of MMP1 (31), a second term was added to favor those *g*_*γ*_ which give smooth distribution of amplitudes across the channels. Spatial smoothness (quantified by Laplacian operators) means basically that the values of 〈*R*^*n*^**x**^*i*^,*g*_*γ*_〉 should be similar for *i* corresponding to the neighboring channels. However, a choice combining two completely different criteria requires some setting of their relative weights. For example, if we attribute too much importance to the spatial criterion, in favor of the energetic one, we may obtain atoms giving very smooth scalp distributions across electrodes. But in such a case the convergence of the MMP procedure, measured in the rate-distortion sense, relating to the amount of explained energy, may be severely impaired. Up to now, no objective or optimal settings for regulating the influence of such extra criteria on the MMP algorithms was proposed.

Finally, we should also point out some of the possible novel and promptly implementable applications of MMP. Variants of the multivariate algorithms, described in section *Multivariate matching pursuit (MMP)*, can be related to several models of multichannel recordings of repetitive trials of evoked brains activity. Algorithms developed for parameterization of EEG structures in subsequent channels [22,24] have been be applied to decomposing subsequent trials of event-related potentials [28,29].

Ongoing works include application of MMP3 to evoked potentials, where variable phase accounts for the variable latencies, and instantaneous decomposition of both repetitions and channels of event-related potentials, with some of the discussed constraints applied separately to the relevant dimensions.

Apart from that, MMP3 may be also used to compute estimates of the phase locking factor [50] (also called inter-trial coherence, [51]). Simultaneous decomposition of all the repetitions will be crucial in this case: in separate MP decompositions of subsequent trials, atoms representing possibly the same structures can have slightly different frequencies, which makes their relative phase insignificant. By estimating the phase coherence only in those are of the time-frequency plane, where indeed an oscillatory activity appears, we may get rid of a lot of noise blurring previously applied estimates.

## Software availability and requirements

Software package described in this article is freely available from http://braintech.pl/svarog/. It can be run on a computer with a reasonably recent version of MS Windows, Mac OS or GNU/Linux with Java runtime environment. Screencasts (video files), showing (1) downloading and configuration of the package and (2) MP decomposition process of a sample EEG epoch via Svarog, are included as [Additional file 1] and [Additional file 2]. These videos can be also viewed in a variety of formats embedded in HTML5 at http://braintech.pl/svarog. [Additional file 3] contains a snapshot of Svarog’s help related to mp5.

Complete source code for the MMP engine written in C is available from http://git.braintech.pl/matching-pursuit.git. GUI is implemented in Java within the Svarog system, for which the source code is available at http://git.braintech.pl/svarog.git. Both projects are released on terms of the General Public License (GPL).

Svarog is a loose acronym for Signal Viewer, Analyzer and Recorder on GPL, and constitutes the core of the world’s first professional EEG recording and analysis system based entirely on GPL software (http://braintech.pl/Manifesto.html). This multiplatform system is developped primarily for GNU/Linux. Current versions of the system, including the above discussed software plus the OpenBCI framework for brain-computer interfaces and a modified version of the PsychoPy framework for experiments design, are available as packages for Ubuntu and Debian (see http://deb.braintech.pl).

## Appendix

### Implementation and optimizations

#### Product update formula

This optimization was proposed in [39].

Let us recall from (1) the formula for the *n*th residuum:

$$R^{n}x=\langle R^{n}x,g_{\gamma_{n}}\rangle g_{\gamma_{n}}+R^{n+1}x$$

Taking the product of both sides with $g_{\gamma_{i}}$, which is the candidate for selection in the next iteration, we get

(38) $$\langle R^{n+1}x,g_{\gamma_{i}}\rangle =\langle R^{n}x,g_{\gamma_{i}}\rangle -\langle R^{n}x,g_{\gamma_{n}}\rangle \langle g_{\gamma_{n}},g_{\gamma_{i}}\rangle$$

This equation expresses the product of a dictionary function $g_{\gamma_{i}}$ with the residuum in step *n*+1 using two products, which were already calculated in the previous iteration—$\langle R^{n}x,g_{\gamma_{i}}\rangle$ and $\langle R^{n}x,g_{\gamma_{n}}\rangle$—and a product of two functions from the dictionary—$\langle g_{\gamma_{n}},g_{\gamma_{i}}\rangle$. Therefore, the only thing that remains to be computed is a product of two known functions.

Inner product of continuous Gabor functions can be expressed in terms of elementary functions (see [47,52]). Unfortunately, it does not reflect with enough accuracy the numerical value of the product of two discrete vectors, representing sampled versions of the same Gabor functions. Exact formula for the product of the latter involves theta functions, which can be approximated by relatively fast converging series [47].

#### Sin, Cos, and Exp: fast calculations and tables

In spite of the trick from the previous section, still—at least in the first iteration—we need to compute the “plain” inner products of the signal with Gabor functions. Using the result from section *Optimal phase of a Gabor function*, of all the phases *ϕ* we calculate products only for *ϕ*=0 and $\phi =\frac{\pi }{2}$.

Computationally, the most expensive part is not the actual calculation of the inner products, but the generation of discrete vectors of samples from Equation (3), which contains cosine and exponent. Compilers usually approximate these functions by high-order polynomials. Although contemporary CPUs may implement directly some special functions, they will still be much more expensive to compute than basic additions or multiplications. Therefore, avoiding explicit calls of these functions may result in significant acceleration—together with tabularization, it accelerated the MP implementation [45] by over an order of magnitude. In the following we show (after [53]) how to fill in a vector with values of sines and cosines for equally spaced arguments using only one call of these functions.

Since the time *t* in (3) in the actual computations is discrete, the trick is to compute sin(*ω*(*t*+1)) knowing sin(*ωt*). Using the trigonometric identity (59) with its corresponding form for the sine function, we get

(39) $$\begin{array}{l} \sin(\omega (t+1))=\sin(\mathrm{\omega t}+\omega )=\cos(\mathrm{\omega t})\sin(\omega )+\sin(\mathrm{\omega t})\cos(\omega ) \end{array}$$

(40) $$\begin{array}{l} \cos(\omega (t+1))=\cos(\mathrm{\omega t}+\omega )=\cos(\mathrm{\omega t})\cos(\omega )-\sin(\mathrm{\omega t})\sin(\omega ) \end{array}$$

We start with *t* = 0, setting cos(0) = 1 and sin(0) = 0, and computing constants cos(*ω*) and sin(*ω*). Values of (39) and (40) for subsequent *t* can be filled in a recursive way, using the computed cos(*ω*) and sin(*ω*) and taking as sin(*ωt*) and cos(*ωt*) values from the previous steps.

A similar approach can accelerate computation of the factors $e^{-\alpha t^{2}}$ present in (3):

(41) $$e^{-\alpha {\left(t+1\right)}^{2}}=e^{-\alpha t^{2}-2\mathrm{\alpha t}-\alpha }=e^{-\alpha t^{2}}e^{-2\mathrm{\alpha t}}e^{-\alpha }$$

To compute (41) we need $e^{-\alpha t^{2}}$ from the previous iteration, constant *e*^−*α*^ independent of *t*, and *e*^−2*αt*^. The last factor can be updated in each iteration at a cost of one multiplication: to get *e*^−2*α*(*t*+1)^ from *e*^−2*αt*^ we multiply it by a precomputed constant *e*^−2*α*^.

In all these cases we also take into account the symmetries sin(−*x*)=− sin(*x*), cos(−*x*)= cos(*x*), and $e^{-{\left(-x\right)}^{2}}=e^{-x^{2}}$ to double the savings. Values of these vectors can be stored in memory for subsequent calculations of Gabor vectors (3) with different combinations of sin/ cos and exp, but only if we restrict the discretization of parameters to some integer grid, for example:

(42) u=1…N,ω=(1…N)π/N,s=1…N

Apart from fast Sin, Cos and Exp function generation, the optimal dictionary allows for saving in the computer memory the tables with values of these functions. The number *N*_*s*_ of different scales in an optimal dictionary is (see Equation (27)):

(43) $$N_{s}=\lfloor \underset{a}{\log}N\rfloor$$

where *N* is the number of samples in signal and *a* is the parameter expressed by Equation (27). A typical epoch of EEG/MEG contains some thousands samples, so it is possible to store all EXP functions in computer memory. Due to the fact, that Gabors, for given scale, are arranged in frequency domain in increments of Δ*ω* (29) one can save also in computer’s memory one period of Sine/Cosine signal of the lowest frequency. The sine and cosine signal of higher frequencies, for example *k*×Δ*ω*, where *k* is the natural number, can be generated by means of selecting every *k*-th sample from Cos/Sine signal of frequency Δ*ω* stored in the computer’s memory.

#### Fast detection of two orthogonal Gabors

Update formula (section *Product update formula*) in combination with optimal dictionary allows for uses the next numerical optimization—fast assessment of orthogonality of two Gabors functions. Let us analyze the analytical formula for an inner product of two Gabors. After substituting to the Equation (18) the exact expression for normalization factor *K*_*γ*_ and constant *C*, and introducing the following factors:

(44) $$\begin{array}{ll} X= & \left(\cos\left[\left(\omega_{0}+\omega_{1})B+(\phi_{0}+\phi_{1})-(\omega_{0}u_{0}+\omega_{1}u_{1}\right)\right]e^{-\pi \frac{{\left(\omega_{0}+\omega_{1}\right)}^{2}}{4A}}\right)\\ & +\left(\cos\left[\left(\omega_{0}-\omega_{1})B+(\phi_{0}-\phi_{1})-(\omega_{0}u_{0}-\omega_{1}u_{1}\right)\right]e^{-\pi \frac{{\left(\omega_{0}-\omega_{1}\right)}^{2}}{4A}}\right)\\ Y= & K_{0}K_{1}\sqrt{s_{1}s_{2}}=\frac{2^{6/4}}{\sqrt{\left(1+\cos(2\phi_{0})e^{-\frac{s_{0}^{2}\omega_{0}^{2}}{2\pi }}\right)\left(1+\cos(2\phi_{1})e^{-\frac{s_{1}^{2}\omega_{1}^{2}}{2\pi }}\right)}}\\ Z= & \sqrt{\frac{\pi }{4A}}{\left(s_{1}s_{2}\right)}^{-\frac{1}{2}}e^{C}=\sqrt{\frac{s_{0}s_{1}}{4\left(s_{0}^{2}+s_{1}^{2}\right)}}e^{-\pi \frac{{\left(u_{0}-u_{1}\right)}^{2}}{s_{0}^{2}+s_{1}^{2}}} \end{array}$$

one can obtain:

(45) $$\langle g_{0},g_{1}\rangle =X\cdot Y\cdot Z$$

It is straightforward to estimate that value of factor *X* fulfils the condition

(46) |X|<2

for every possible set of parameters {*s*_0_,*s*_1_,*ω*_0_,*ω*_1_,*ϕ*_0_,*ϕ*_1_,*u*_0_,*u*_1_}. The maximal value of parameter *Y* is limited by the lowest values of scale *s* and frequency *ω*, which, based on (29), are:

(47) $$\begin{array}{l} \begin{array}{ll} s & =s_{\text{min}}>0\\ \omega & =\Delta \omega =\frac{2\sqrt{\pi }}{s_{\text{min}}}\sqrt{-\log(1-\epsilon^{2})} \end{array} \end{array}$$

and phases $\phi_{0}=\phi_{1}=\pm \frac{\pi }{2}$. Substituting above formulae into factor *Y* results in following expression:

(48) $$|Y|\leq \frac{2^{3/2}}{\sqrt{\left(1+\cos(2\phi_{0}){\left(1-\epsilon^{2}\right)}^{2}\right)\left(1+\cos(2\phi_{1}){\left(1-\epsilon^{2}\right)}^{2}\right)}}\leq \frac{2^{3/2}}{\epsilon^{2}(2-\epsilon^{2})}\approx \frac{\sqrt{2}}{\epsilon^{2}}$$

Based on this observation, it is possible to introduce a numerical threshold, defining an approximate orthogonality of two Gabor functions. The factor *Z* depends on the relative position and the width of two Gabors. Atoms which differ mostly in these parameters will give a small factor *Z*. Therefore

(49) $$\frac{2\sqrt{2}}{\epsilon^{2}}Z<\eta \Rightarrow |\text{XYZ}|<\eta \Rightarrow \langle g_{0},g_{1}\rangle \approx 0$$

where *η* can be set for example at the accuracy of a double precision number (10^−16^). Condition (49) allows for efficient detection of orthogonal atoms in dictionary and replacing their inner product by zero in Equation (38). Moreover, in case of a dictionary with uniform step Δ*ω* at a given scale, it is possible to determine the set of Gabor functionss, characterized by the same position *u*, for which inner product with the Gabor selected in previous iteration will be zero.

#### Limiting domain of the product

When Equation (49) is not fulfilled, the two Gabor functions cannot be treated as orthogonal, and their product has to be determined. Full scalar product of two Gabor functions *g*_1_ and *g*_2_ can be written as

(50) $$\langle g_{1},g_{2}\rangle =K_{1}K_{2}\int_{-\infty }^{+\infty }e^{-A{\left(t-B\right)}^{2}}e^{C}\cos(\omega_{1}(t-u_{1})+\phi_{1})\cos(\omega_{2}(t-u_{2})+\phi_{2})\;\text{dt}$$

where A, B and C are defined in Equations (15–17).

To perform numerical integration, one can replace the improper integral above with a definite integral on a sufficiently large interval [ *a*;*b*], so that

(51) $$\left|\int_{-\infty }^{+\infty }g_{1}(t)g_{2}(t)\;\text{dt}-\int_{a}^{b}g_{1}(t)g_{2}(t)\;\text{dt}\right|<\eta$$

for given error bound *η*. Such interval can be constructed as [ *B*−Δ;*B*+Δ] to fulfil

(52) $$\begin{array}{l} \begin{array}{l} \left|\int_{B+\Delta }^{+\infty }g_{1}(t)g_{2}(t)\;\text{dt}\right|<\frac{1}{2}\eta \\ \left|\int_{-\infty }^{B-\Delta }g_{1}(t)g_{2}(t)\;\text{dt}\right|<\frac{1}{2}\mathrm{\eta .} \end{array} \end{array}$$

Therefore, Δ can be calculated as

(53) $$\Delta =\frac{1}{\sqrt{A}}\overset{-1}{\mathrm{erf}}\left(1-\frac{\eta \epsilon^{2}}{\sqrt{2}}e^{-C}\sqrt{\frac{s_{1}}{s_{2}}+\frac{s_{2}}{s_{1}}}\;\right),$$

where erf−1 is an inverse of the error function

(54) $$\mathrm{erf}(x)=\frac{2}{\sqrt{\pi }}\int_{0}^{x}e^{-t^{2}}\mathrm{dt.}$$

The value of Δ in (53) is well-defined unless inequality (49) is fulfilled, in which case the atoms are orthogonal to the given precision and there is no need to define integration interval.

To simplify formula (53), one can use inequality

(55) $$\overset{-1}{\mathrm{erf}}(1-x)\leq \sqrt{-\log x},$$

which is fulfilled for all *x*∈(0;1]. Therefore,

(56) $$\Delta^{\prime }=\frac{1}{\sqrt{A}}\sqrt{C-\log\left(\frac{\eta \epsilon^{2}}{\sqrt{2}}\sqrt{\frac{s_{1}}{s_{2}}+\frac{s_{2}}{s_{1}}}\right)}$$

is guaranteed to fulfil Δ^′^ ≥ Δ.

#### Optimal phase of a Gabor function

In the following we find an explicit formula for the phase *ϕ*_max_, that maximizes the product of signal *x* with Gabor function of given time position *u*, frequency *ω*, and scale *s*. Let us recall the formula (3) of a real Gabor function:

$$g_{\gamma }(t)=K(\gamma )e^{-\pi {\left(\frac{t-u}{s}\right)}^{2}}\cos\left(\omega (t-u)+\phi \right).$$

*γ* denotes the set of parameters *γ*={*u*,*s*,*ω*,*ϕ*} and *K*(*γ*) is such that ||*g*_*γ*_||=1. Writing *K*(*γ*) explicitly gives

(57) $$g_{\left(\gamma \right)}(t)=\frac{e^{-\pi {\left(\frac{t-u}{s}\right)}^{2}}\cos\left(\omega (t-u)+\phi \right)}{\left||e^{-\pi {\left(\frac{t-u}{s}\right)}^{2}}\cos\left(\omega (t-u)+\phi \right)|\right|}$$

Phase shift *ϕ* can be also expressed as a superposition of two orthogonal oscillations cos(*ω*(*t*−*u*)+*ϕ*) and sin(*ω*(*t*−*u*)+*ϕ*). We define

(58) $$\begin{array}{l} C=e^{-\pi {\left(\frac{t-u}{s}\right)}^{2}}\cos\left(\omega (t-u)\right)\\ S=e^{-\pi {\left(\frac{t-u}{s}\right)}^{2}}\sin\left(\omega (t-u)\right) \end{array}$$

and, using the trigonometric identity

(59) cos(α+ϕ)=cosαcosϕ−sinαsinϕ,

we write the Gabor function (57) as

(60) $$g_{\gamma }(t)=\frac{C\cos\phi -S\sin\phi }{∥C\cos\phi -S\sin\phi ∥}$$

Using ∥*x*∥^2^=〈*x*,*x*〉, and the orthogonality of *C* and *S* defined in (58) (〈*C*,*S*〉=0), we write the product of Gabor function defined as (60) with the signal *x* as

(61) $$\langle x,g_{\gamma }\rangle =\frac{\langle x,C\rangle \cos\phi -\langle x,S\rangle \sin\phi }{∥C\cos\phi -S\sin\phi ∥}=\frac{\langle x,C\rangle -\langle x,S\rangle \tan\phi }{\sqrt{\langle C,C\rangle +\langle S,S\rangle \overset{2}{\tan}\phi }}$$

We are looking for the maximum absolute value of this product. For the sake of simplicity we will maximize 〈*x*,*g*_*γ*_〉^2^ instead of |〈*x*,*g*_*γ*_〉|. Denoting *v*= tan*ϕ*

(62) $${\langle x,g_{\gamma }\rangle }^{2}=\frac{{\left(\langle x,C\rangle -\langle x,S\rangle v\right)}^{2}}{\langle C,C\rangle +\langle S,S\rangle v^{2}}$$

To find *v* which maximizes (62), we look for zeros of the derivative $\frac{\partial }{\mathrm{\partial v}}{\langle x,g_{\gamma }\rangle }^{2}$ and find two roots:

$$v_{1}=\frac{\langle x,C\rangle }{\langle x,S\rangle },\;\;\;\;v_{2}=-\frac{\langle x,S\rangle \langle C,C\rangle }{\langle x,C\rangle \langle S,S\rangle }$$

Substituting these values for tan*ϕ* in (61) we get

$$\langle x,g_{\gamma }\rangle {|}_{v=v_{1}}=0$$

$$\langle x,g_{\gamma }\rangle {|}_{v=v_{2}}=\frac{\langle x,C\rangle +\frac{{\langle x,S\rangle }^{2}\langle C,C\rangle }{\langle x,C\rangle \langle S,S\rangle }}{\sqrt{\langle C,C\rangle \;+\frac{{\langle x,S\rangle }^{2}{\langle C,C\rangle }^{2}}{{\langle x,C\rangle }^{2}\langle S,S\rangle }}}$$

Since for *v*_1_ the square of the product is minimum (zero), the other extremum is a maximum in *v*_2_. Therefore, the phase *ϕ* that maximizes 〈*x*,*g*_*γ*_〉^2^ is given by

(63) $$\phi_{\max}=\arctan\frac{\langle x,S\rangle /\langle S,S\rangle }{\langle x,C\rangle /\langle C,C\rangle }$$

and the maximum value of the product is

(64) $${\langle x,g_{\gamma }\rangle }_{\max}=\frac{{\langle x,C\rangle }^{2}/\langle C,C\rangle +{\langle x,S\rangle }^{2}/\langle S,S\rangle }{\sqrt{{\langle x,C\rangle }^{2}/\langle C,C\rangle +{\langle x,S\rangle }^{2}/\langle S,S\rangle }}$$

#### Applying Fast Fourier Transform

Estimation of the product of a Gabor function with signal *x* (61) requires computing of the inner product of signal *x* with functions *C* and *S* defined in Equation (58). Let us rewrite the formulae for <*x*,*C*> and <*x*,*S*>:

(65a) $$\langle x,C\rangle =\int_{-\infty }^{\infty }xe^{-\pi {\left(\frac{t-u}{s}\right)}^{2}}\cos\left(\omega (t-u)\right)$$

(65b) $$\langle x,S\rangle =\int_{-\infty }^{\infty }xe^{-\pi {\left(\frac{t-u}{s}\right)}^{2}}\sin\left(\omega (t-u)\right)$$

Multiplying (65a) by $i=\sqrt{-1}$ and substracting it from (65b), one can obtain the following equation:

(66) $$\langle x,C\rangle -i\langle x,S\rangle =\int_{-\infty }^{\infty }xe^{-\pi {\left(\frac{t-u}{s}\right)}^{2}}e^{-\mathrm{i\omega}(t-u)}$$

The right side of Equation (66) is the Fourier Transform of signal *x* windowed by Gauss functions $e^{-\pi {\left(\frac{t-u}{s}\right)}^{2}}$. This formula allows for fast computation of inner products <*x*,*C*> and <*x*,*S*>, since in and optimal dictionary the atoms with the given scale *s* are arranged in frequency domain with uniform step Δ*ω* (see Equation (29)).

#### Additional structures in the dictionary

Apart from the Gabor functions, Gabor dictionary implemented in mp5 contains also the following functions: 

• “Pure” harmonic waves

(67) $$S(t)=K_{s}\cos(\mathrm{\omega t}+\phi )$$

where *K*_*s*_ is normalization factor such that 〈*S*(*t*),*S*(*t*)〉=1 on the analysed signal length. The phase *ϕ* of the signal *S*(*t*) is estimated according to Equation (63).

Harmonic functions are distributed in frequency domain with step Δ*ω* determined by Equation (29);

• Kronecker delta functions

(68) $$\Delta (t-u)=\{\begin{array}{l} 1,\text{for}\;t=u\\ 0,\text{for}\;t\neq u \end{array}$$

In this work, the delta functions are distributed across the whole time domain, that is for each point of the time series.

• “pure” Gaussians

(69) $$G(t)=K_{g}e^{-\pi \frac{{\left(t-u\right)}^{2}}{s^{2}}}$$

Where *K*_*g*_ is normalization factor such that 〈*G*(*t*),*G*(*t*)〉=1 on the analysed signal length. These functions are distributed in the scale domain with step *a* (see Equation (27)) and with step Δ*u* (30) in the time domain.

## Abbreviations

EEG: Electroencephalogram; FFT: Fast Fourier Transform; MEG: Magnetoencephalogram; MP: Matching pursuit; MMP: Multivariate matching pursuit; S/N: Signal to noise.

## Competing interests

The authors declare that they have no competing interests.

## Authors’ contributions

RK wrote from the scratch the the mp5 implementation in C, and contributed most of the text in Appendix 1. PTR derived the formulae for product-related metric and resulting dictionary construction, adapted Svarog and its GUI to the modifications in the algorithm, and wrote most of the sections introducing optimal sampling of Gabor dictionaries. PJD proposed the idea of optimal sampling of Gabor dictionaries based upon the product-related metrics in 2003, over the next decade supervised and coordinated projects which led to this article and the accompanying software, and wrote the remaining text. All authors read and approved the final manuscript.

## Supplementary Material

Additional file 1 A video tutorial for downloading and configuration of the Svarog package, used for computations and visualization of results. Click here for file

Additional file 2 Shows the steps from loading the signal into Svarog to displaying interactive map of time-frequency energy distribution. Click here for file

Additional file 3 Contains help of the MP module from Svarog. Click here for file
