# Supplementary material for: Multivariate matching pursuit in optimal Gabor dictionaries: theory and software with interface for EEG/MEG via Svarog
Source: Biomed Eng Online. 2013 Sep 23;12:94. doi: 10.1186/1475-925X-12-94 (PMC3849619; doi:10.1186/1475-925X-12-94)
Supplement: Additional file 3 — Contains help of the MP module from Svarog. [file 1475-925X-12-94-S3.zip › index.html]

mp5 help


# Matching pursuit decomposition in Svarog

**Mp5** software for matching pursuit (MP)
decomposition of (biomedical multivariate) time series was
developed at the University of
Warsaw, Faculty of Physics.
The algorithm is based upon the seminal paper by Mallat and Zhang (Mallat1993) with modifications
described partly by Durka et al. (Durka2001) and Kuś et al. (Kuś2013). Mp5 is designed
for batch processing input signals and writing results to disk
files called—after (Mallat1993)—*decomposition
books*. These books (**\***.b) contain
parameters of the functions selected for representation of the
analyzed signal.

Svarog offers an interactive interface for MP decomposition,
allowing for:

1. simple selection of the part of the signal for MP
   decomposition,
2. running mp5 decomposition according to selected parameters
3. visualization of the results in terms of interactive
   time-frequency maps of signal's energy density.

Some of the topics presented below are covered in a screencast
avaialble at http://braintech.pl/svarog/screencast.html. *Caveat*: default settings are optimized for speed rather
than quality of the decomposition. This may be sufficient for the
first try, but serious use requires some understanding of the
procedure.

Contents:

1. Configuration
2. Selecting the signal epoch(s)
   for decomposition
3. Detailed settings and structure
   of the mp5 config file
4. Comments in the config file
5. Input settings — obligatory
   settings
   1. nameOfDataFile
   2. nameOfOutputDirectory
   3. writingMode
   4. numberOfChannels
   5. selectedChannels
   6. numberOfSamplesInEpoch
   7. selectedEpochs
   8. typeOfDictionary
   9. energyError
   10. randomSeed
   11. reinitDictionary
   12. maximalNumberOfIterations
   13. energyPercent
   14. MP
   15. scaleToPeriodFactor
   16. samplingFrequency
   17. pointsPerMicrovolt- Input parameters — additional
     settings
     1. normType
     2. diracInDictionary
     3. gaussInDictionary
     4. sinCosInDictionary
     5. gaborInDictionary
     6. progressBar
   - Example configuration file
   - References

## Configuration

By default, Svarog looks for the file containing the mp5 binary
in PATH. If you installed the system from .deb packages (see http://deb.braintech.pl) it
should be already preconfigured. If that does not work, you should
provide the correct path in the "Preferences" dialog (`Edit/Preferences`),
tab `Tools`:

If you downloaded mp5 together with Svarog in one archive,
executables for different operating systems can be located under `mp5` in subfolders `linux`, `windows`, and `mac`.

## Selecting the signal epoch(s) for decomposition

Using icons in the toolbar, mark in Svarog the part of the signal
that you want to decompose and select `Tools/MP decomposition` from the menu

`Signal selection` tab allows you to change selection or e.g. set
up decomposition of the whole signal page by page. Settings in
other tabs correspond to the parameters of decomposition that will
be written to the configuration file. Their meaning is explained
in the next chapter.

After pressing `Ok` we get the window showing the progress of the
task

Execution time depends strongly on selected settings and length
of the signal being decomposed. It can be quite long for
multichannel decompositions. If a single channel was selected (as
in the picture above) with default settings, and the sampling
frequency was not extremely high (that is, there are no more than
few thousand points in the selected epoch) decomposition should
not take more than a minute on average PC.

You can move this window aside and continue working with Svarog
in the meantime.

After the task completes, `Get result` button will
appear at the bottom of the window. Pressing it opens a dialog
where you can choose `Open in the viewer` and/or `Save
to disk`. The former option opens a new tab in Svarog and
diplays the timefrequency map of the signal's energy density,
computed from the decomposition:

  

Below the map the program displays the original signal, its
reconstruction from all the atoms from the (just computed)
decomposition, and the reconstruction computed from the selected
atoms. You can select atoms by clicking the crosses in their
centers. After clicking the magnifying glass in the top left, you
can zoom selected area of the map.

# Detailed settings and structure of the mp5 config file

Parameters of the decomposition, entered in subsequent tabs of
the `MP Decomposition configuration` window, are
written to a configuration file, which is in turn passed by Svarog
to the mp5 binary. Mp5 config file consists of lines, which can be
divided into three groups:

- Comments.
- Obligatory settings.
- Additional settings.

Each line, which is not a comment, is treated as a setting for
the program. Each setting consists of its name and assigned value.
If any of the obligatory settings is missing, mp5 will return
error. If the user does not set the additional settings, mp5 sets
the default values. Lines with commands and comments can occur
within the config file in any order.

## Comments in the config file

The user can add his own comments to the configuration file by
placing a # sign in front of the line. There are two type of
comments:

- lines starting with # are treated as comments and will exist
  only in the config file, but will be neglected by mp5, for
  example:  
  `# Moly set the numberOfChannels to 5`
- lines starting with ## are also treated as comments but they
  will be copied into the output file (decomposition book), e.g.:  
  `## channels selected above motor cortex decomposed with
  very small dictionary`

Although in general the results are written to the binary file,
the comments are saved as text and can be viewed by standard text
editor.

## Input settings — obligatory settings

Signal (time series) for mp5 decomposition must be stored
in a binary file as a 4-byte float numbers.

In case of multivariate
(multichannel) recordings the values should be multiplexed, that is:  
`s1ch1, s1ch2, …, s1chK, s2ch1, s2ch2, …, s2chK`  
where `sXchY` is the X-th sample at channel Y, K is the
number of channels

### nameOfDataFile

Full path to the input file.

### nameOfOutputDirectory

The path to the output directory, where the file with results
should be saved. The default is the same as location of the input
file.

### writingMode

- `CREATE` — create a new file for writing (saving results of decomposition).
- `APPEND` — append the results to an already
  existing file or create a new file if there is no file to append
  to

Names of the output files are generated automatically; the name
of the file with the data is appended with: `_smp.b` in case of a single channel mp algorithm (SMP), or `_mmp.b` in case of one of the multichannel mp algorithms (MMP1, MMP2,
MMP3). The decomposition book includes also the original analyzed
epoch.

### numberOfChannels

Number of channels in the input file. Positive integer,
range: 1 - 65535.

### selectedChannels

In either of the algorithms—monochannel or multivariate—one does
not have to use all the channels present in the data file. For
example, using SMP we may decompose only few channels, and for MMP
we may need to neglect non-EEG channels. This option lists the
channels which we want to analyze. That is, to analyze all the
channels from a 20-channel datafile config must contain:

```
numberOfChannels 20
selectedChannels 1-20
```

Channels are numbered starting from 1. Selection is written
either as comma-separated list 1,2,3 or as ranges 1-3. Both can be
combined in one line, e.g.:

```
selectedChannels 1, 3, 5, 7-11, 19
```

type: positive integer,   
range: 1 - 65535

### numberOfSamplesInEpoch

When decomposing susequent epochs of a long signal, this option
determines the length of the epoch measured as number of samples.
Argument's value: positive integer. Range: 1 - 2147483647

### selectedEpochs

Chooses which epochs (of the length defined by `numberOfSamplesInEpoch`)
will be analysed. First epoch in the file is number 1. Written as
comma-separated list with ranges as in `selectedChannels`.

### typeOfDictionary

- `OCTAVE_FIXED` — functions distributed in a way to
  optimally cover the ranges of parameters reasonable for a given
  signal in a way that the distance between any two neighboring
  functions does not exceed the threshold given by a user (Kuś et al. 2013)
- `OCTAVE_STOCH` — distribution as in `OCTAVE_FIXED`,
  plus a stochastic element to remove the possible bias (Durka et al. 2001). The dictionary
  with Gabors is created according to the first parameter
  energyError command, but in the next step a fraction of randomly
  selected functions are removed from dictionary. The number of
  atoms left is controlled by the second value passed to the  `EnergyEr``ror` parameter.

### energyError

This parameter regulates the density of the dictionary. That is,
for the same energyError, and different sizes of the analyzed
epoch, effective sizes of the dictionary will be larger for longer
epochs, but accuracy of the decomposition should be equivalent
(except for the border effects). Changing this parameter in the
"Basic settings" tab automatically computes the amount of RAM
necessary to store the dictionary that will be prepared for
decomposition.

The energyError parameter has two values:

- the first one is a threshold for a distance between two
  nearest atom in dictionary, this value should be in range (0 1)
- the second is value percentage of atoms left after stochastic
  dictionary reduction; this value is omitted by mp5, when
  typeOfDictionary is set to OCTAVE\_FIXED

For example, the configuration of commands:

```
typeOfDictionary OCTAVE_FIXED
energyError 0.3 30.0
```

results in generation of dictionary in which the maximal distance
between nearest Gabors does not exceed value 0.3.

With another configuration:

```
typeOfDictionary OCTAVE_STOCH
energyError 0.3 40.0
```

the distribution of the atoms in dictionary is performed in such
way that the maximal distance between nearest Gabors does not
exceed value 0.3. After generation of the dictionary, the number
of Gabors will be reduced to 40% of their initial amount
(determined by the threshold 0.3) by random selection.

type of the first parameter (energyError): float number  
range of the first parameter: (0.0 1.0)

type of the second parameter (stochastic reduction): float number  
range of the second parameter: (0.0 100.0>

### randomSeed

If provided, this value will be used for the seed of the random
generator used in creations a stochastic dictionary. Otherwise,
the seed will be generated from the computer's clock.

For example:

```
randomSeed 23432
```

The mp5 will used this number as a seed for random generator. The *seed* is the same for any stochastic reinitialization of the
dictionary (see the next command: reinitDictionary).

Second example:

```
randomSeed auto
```

The mp5 will used the time form the computer's clock as a seed
for therandom generator. The seed is different for each
reinitialization of the dictionary.

Type: string "auto" or positive integer number  
range in case of integer: 0 - 2147483647

### reinitDictionary

When using the default stochastic (randomized) dictionary, the
randomization is called reinitialization of the dictionary. It can
be performed:

- `REINIT_AT_ALL` — at each of the analyzed epochs,
  works only for `SMP`.
- `REINIT_IN_CHANNEL_DOMAIN` — exactly the same
  dictionary will be used to decompose all epochs within a
  channel. Before the analysis of another channel, parameters will
  be reinitialized, i.e. randomized again. This setting can be
  applied only in the case of separate decomposition of channels
  using `SMP` option.
- `REINIT_IN_OFFSET_DOMAIN` — dictionary will be
  reinitialized before decomposing each subsequent epoch.
- `NO_REINIT_AT_ALL` — the same dictionary for
  decompositions in all channels and all epochs.

### maximalNumberOfIterations

Maximum number of iterations equals the largest allowed numbers
of waveforms fitted to the analyzed epoch. This command together
with the next `energyPercent` control the number of
waveforms used for the approximation of the analyzed epoch, which
equals the number of algorithms iteration performed before the
stopping criterion is fulfilled. The iterations stop either after
reaching this number, or after explaining the percentage of
signal's energy set in the `energyPercent` parameter,
whatever comes first. That is, if we want to force the
decomposition to contain exactly `maximalNumberOfIterations` iterations, we should set `energyPercent` to 100. It
does not influence the accuracy of the parameters of the waveforms
fitted to the signal, in the sense that the 5 waveforms
fitted to the signal in 5 iterations run will be just as good as
the first 5 fitted in the 100 iterations run.

Time of computations is proportional to the number of algoritms
iterations (except for the first, longer iteration), and obviously
with more iterations we explain larger fraction of signal's
energy; OTOH, too many iterations usually make no sense.
Unfortunately, the meaning of "too many" depends on the properties
of the signal and the aim of decomposition. For further
considerations consult e.g. (Durka2007a) and (Durka2007b).

Type: positive integer  
range: <1 65535>

### energyPercent

Algorithm stops after explaining `energyPercent` %
of the energy of the analyzed epoch, that is, when the sum of
energies of functions fitted to the signals reaches `energyPercent`/100
of the signal's energy, unless `maximalNumberOfIterations` was reached first.

Type: positive float  
range: (0 100.0)

### MP

There are significant differences between the 'classical' MP
computed for the univariate time series, and the variety of
possible versions of the multichannel MP. Choice of the
appropriate flavor of multivariate algorithm is controlled by the
MP variable, which can be assigned one of the following values.
For equations and formulae see (Durka 2007a) and (Kuś et al. 2013)

- `SMP` — monochannel MP (separate decomposition of
  single channels)
- `MMP1` — multichannel algorithm maximizing in each
  iteration the sum of energies explained in all channels
- `MMP2` — suboptimal, faster version of `MMP1` maximizing in each iteration the sum of products of the waveform
  with all the channels. In theory may degrade in the case when
  opposite polarities are present across channels
- `MMP3` — as `MMP1`, but allowing
  different phases in each channel.

### scaleToPeriodFactor

All Gabor functions, for which the number of periods of the
sine, given by this parameter, exceeds the half-width of the
Gaussian, will be removed from the dictionary. It regulates
removal of non-physical cases. For example, 1 Hz oscillation
which lasts 100 milliseconds mathematically represents a well
defined waveform. However, the very notion of frequency is
dubious in such case. Also, the actul peak-to-peak amplitude of
such function may significantly differ from the doubled amplitude
of the Gaussian envelope. See (Durka2004)

Type: positive float  
range: (0.0 3.402823466 E + 38>

### samplingFrequency

Floating-point positive number. Decimal dot is obligatory, that
is "`128.0`" and not "`128`". This parameter
has no influence on the decomposition -- value is copied into the
decomposition book file and can be later used for scaling the
display of results.

### pointsPerMicrovolt

Conversion rate from the actual values stored in the datafile to
physical units, for EEG microvolts [µV], for MEG femtotesla [fT].
This parameter has no influence on the decomposition -- value is
copied into the decomposition book file and can be later used for
scaling the display of results.

Decimal dot is obligatory. For example,

```
pointsPerMicrovolt 20.0
```

in EEG file says that the stored vaules must be multiplied by 20
to get microvolts.

Type: positive float  
range: (0.0 3.402823466 E + 38>

## Input parameters — additional settings

### normType

The experimental feature of mp5 program, do not change it. The
default value is set to `L2`.

### diracInDictionary

Generate dictionary including Dirac Function. The possible
argument of command is `YES` or `NO` string.

### gaussInDictionary

Generate dictionary including Gauss Function. The possible
argument of command is `YES` or `NO` string.

### sinCosInDictionary

Generate dictionary including Harmonic function. The possible
argument of command is `YES` or `NO` string.

### gaborInDictionary

Generate dictionary including Gabor functions. The possible
argument of command is `YES` or `NO` string.

### progressBar

Plot text bar representing the progress of calculation. The
possible argument of command is `YES` or `NO` string.

# Example configuration file

```
# OBLIGATORY PARAMETERS
nameOfDataFile         		test.dat
nameOfOutputDirectory  		./
writingMode            		CREATE
samplingFrequency      		128.0
numberOfChannels       		24
selectedChannels       		1-19
numberOfSamplesInEpoch 		179
selectedEpochs         		1-31
typeOfDictionary       		OCTAVE_FIXED
energyError            		0.3 50.0
randomSeed             		auto
reinitDictionary       		NO_REINIT_AT_ALL
maximalNumberOfIterations 	100
energyPercent             	95.0
MP                        	SMP
scaleToPeriodFactor       	1.0
pointsPerMicrovolt        	1.0

# ADDITIONAL PARAMETERS
normType               		L2
diracInDictionary       	YES
gaussInDictionary       	YES
sinCosInDictionary     	   	YES
gaborInDictionary      	   	YES
progressBar            	   	ON
```

---

# References

(Mallat1993) Stéphane Mallat and
Zhifeng Zhang  
Matching pursuit with time-frequency dictionaries. IEEE Transactions on Signal
Processing 1993, 41:3397-3415

(Durka2007a) Piotr J. Durka  
Matching
Pursuit and Unification in EEG analysis, Artech House 2007,
ISBN 978-1-58053-304-1

(Durka2007b) Piotr J. Durka  
Matching
Pursuit, Scholarpedia, p. 20910, 2007.

(Kuś et al.2013) Rafał Kuś, Piotr T.
Różański and Piotr J. Durka  
Multivariate matching pursuit in optimal Gabor dictionaries:
mathematical foundations and software for EEG/MEG (submitted to Biomedical Engineering Online)

(Durka et al. 2001) Piotr J. Durka, D.
Ircha and K. J. Blinowska  
Stochastic time-frequency dictionaries for Matching Pursuit, IEEE Transactions on Signal
Processing, vol. 49, No. 3, pp. 507-510, March 2001.

(Durka2004) Adaptive time-frequency
parametrization of epileptic EEG spikes P.J. Durka, Physical
Review E, vol. 69, 051914 (2004)
