# Supplementary figures and images for: Multivariate matching pursuit in optimal Gabor dictionaries: theory and software with interface for EEG/MEG via Svarog
Source: Biomed Eng Online. 2013 Sep 23;12:94. doi: 10.1186/1475-925X-12-94 (PMC3849619; doi:10.1186/1475-925X-12-94)

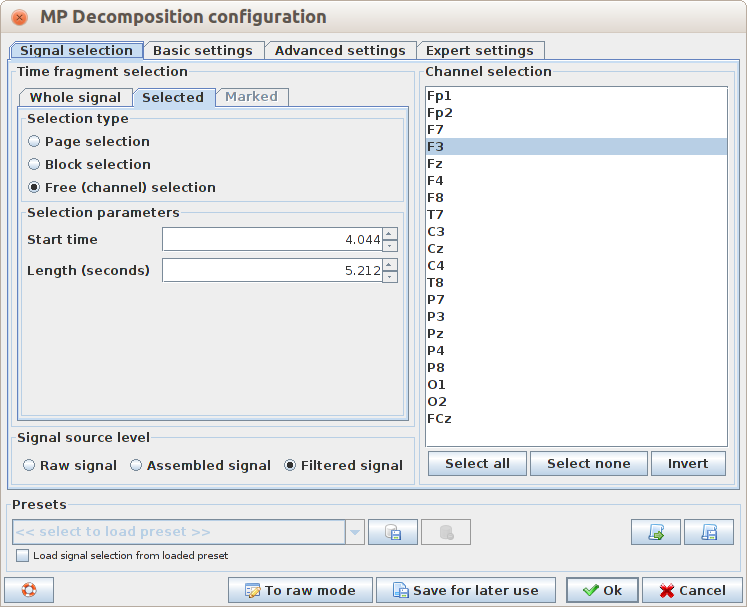

Supplement: Additional file 3 — Contains help of the MP module from Svarog. [file 1475-925X-12-94-S3.zip › MP_Decomposition_configuration.png]

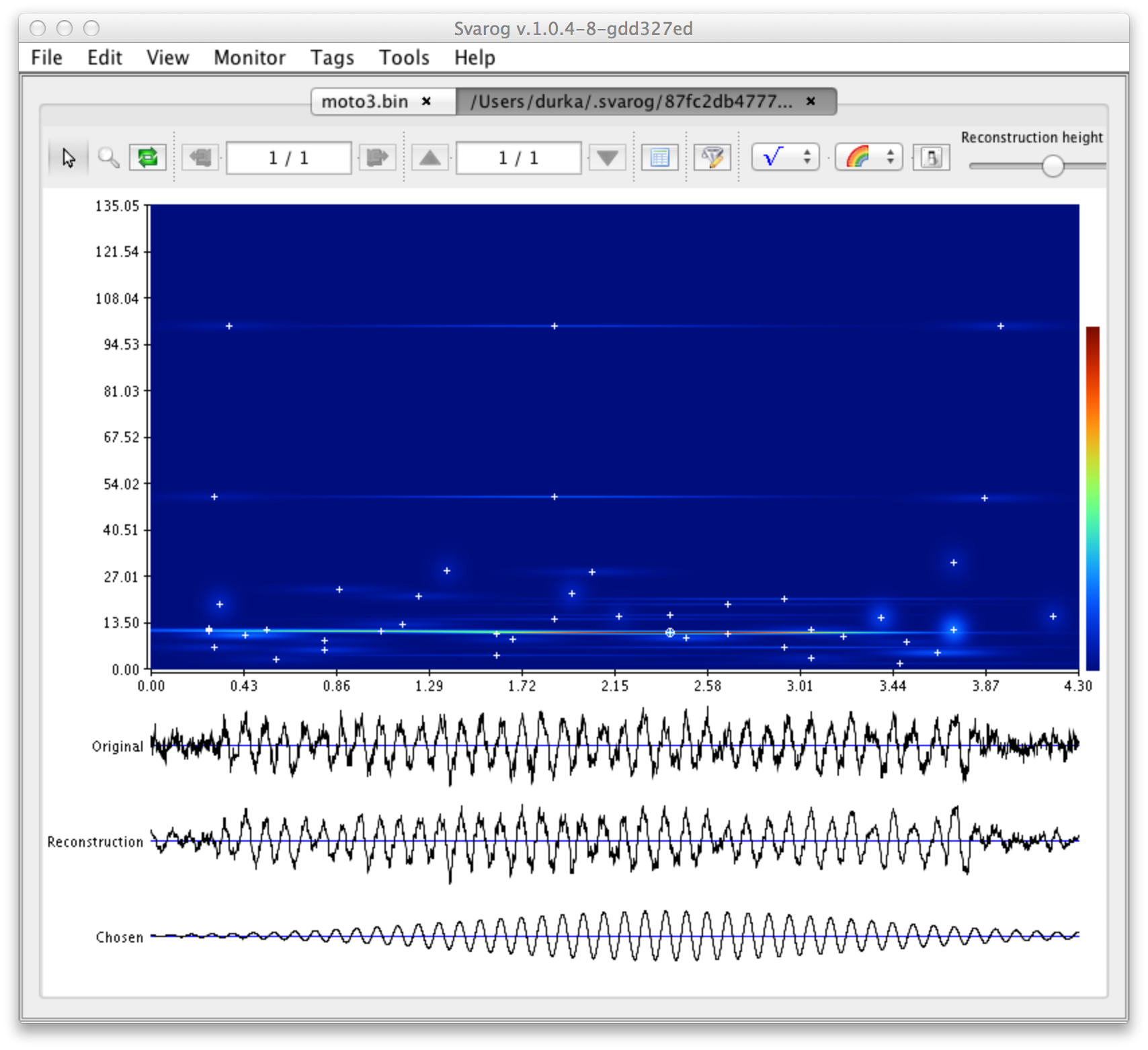

Supplement: Additional file 3 — Contains help of the MP module from Svarog. [file 1475-925X-12-94-S3.zip › Svarog_MP_book_zoom_select.png]

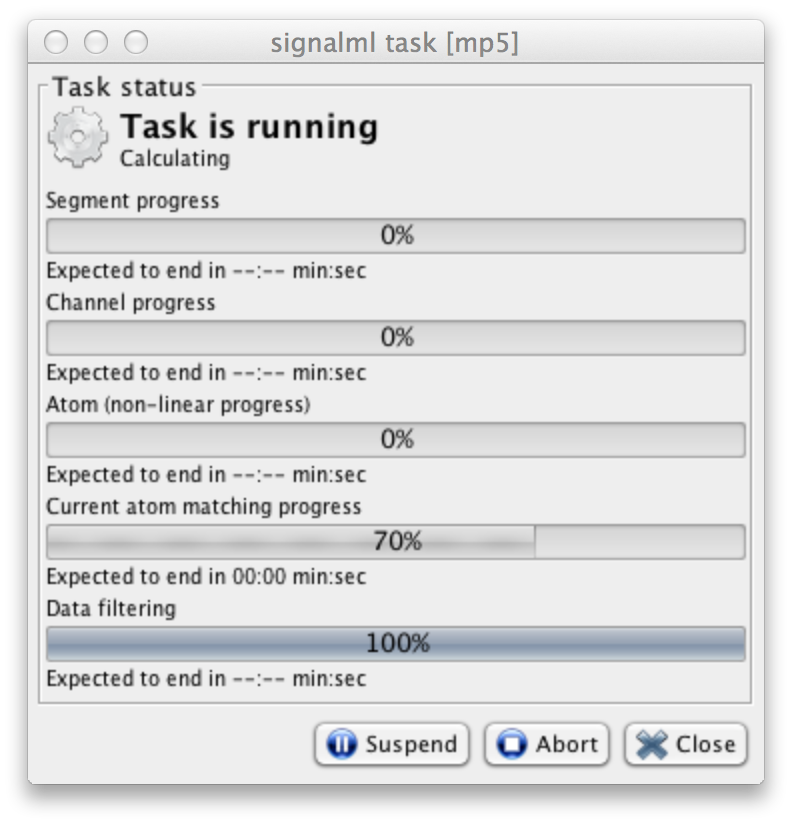

Supplement: Additional file 3 — Contains help of the MP module from Svarog. [file 1475-925X-12-94-S3.zip › Svarog_MP_progress.png]

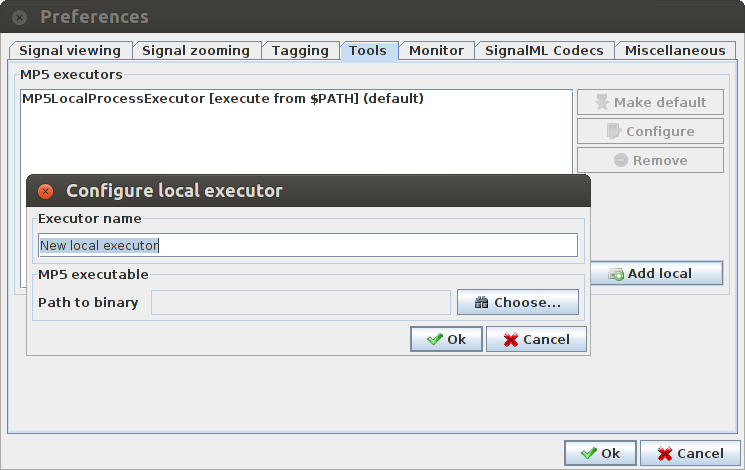

Supplement: Additional file 3 — Contains help of the MP module from Svarog. [file 1475-925X-12-94-S3.zip › Svarog_choose_MP_executor.png]

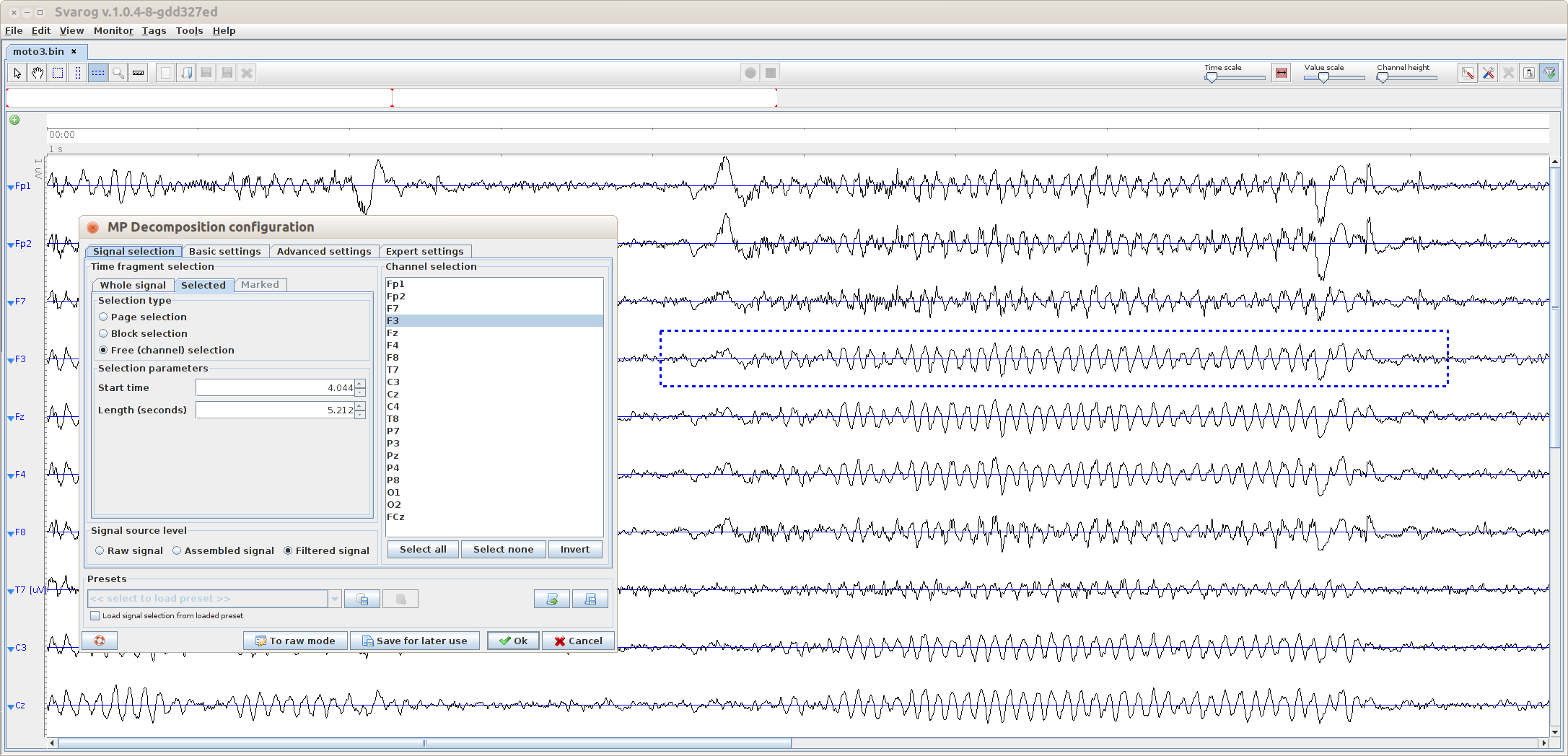

Supplement: Additional file 3 — Contains help of the MP module from Svarog. [file 1475-925X-12-94-S3.zip › Svarog_signal_selection_MP.png]
